# TMED9: a potential therapeutic target and prognostic marker in glioma and its implications across pan-cancer contexts

**DOI:** 10.3389/fimmu.2025.1558881

**Published:** 2025-03-07

**Authors:** Benyong Mi, Chaolin Li

**Affiliations:** Department of Pediatrics, Jinniu District Maternal and Child Health Hospital, Chengdu, China

**Keywords:** TMED9, glioma, immunity therapy, EMT, biomarkers

## Abstract

**Background:**

The escalating global cancer burden, projected to reach 35 million new cases by 2050, underscores the urgent need for innovative cancer biomarkers to improve treatment efficacy and patient outcomes. The TMED family, particularly TMED9, has garnered attention for its involvement in cancer progression; however, its comprehensive role across various cancer types remains poorly understood.

**Methods:**

Utilizing multi-omics data, we analyzed the expression pattern, prognostic significance, genomic alterations, and immunological features of TMED9 in various cancer types. Through *in vitro* experiments, we paid special attention to its role in glioma, especially its correlation with glioma cell migration and invasion behavior.

**Results:**

Our findings reveal that TMED9 is significantly overexpressed in various tumor tissues and is associated with poor prognosis in cancers such as glioblastoma and lower-grade gliomas. Genetic analysis shows TMED9 mutations predominantly in kidney renal clear cell carcinoma, with its expression linked to chromosomal instability. Immunological analysis indicates that TMED9 correlates positively with immune cell infiltration, particularly macrophages, suggesting its role in promoting tumor immunity. Furthermore, TMED9 expression was negatively correlated with tumor stemness, indicating its potential influence on chemotherapy resistance. Knockdown of TMED9 led to reduced migration and invasion in glioma cell lines.

**Conclusions:**

Our comprehensive analysis positions TMED9 as a critical player in cancer progression and immune modulation, especially in gliomas. Elevated TMED9 expression correlates with poorer outcomes and may serve as a prognostic marker and therapeutic target. Future research should focus on elucidating TMED9’s mechanistic pathways and validating its role in clinical settings to enhance glioma treatment strategies.

## 1 Introduction

The global cancer burden is increasing. In 2022, there were nearly 20 million new cancer cases and 9.7 million cancer deaths worldwide. It is estimated that by 2050, the number of new cancer cases will reach 35 million (1). Although antibodies targeting inhibitory immune checkpoints and CAR-T cell therapy have shown promise in various malignancies, including melanoma, non-small cell lung cancer, and renal cell carcinoma, patient specificity, tumor heterogeneity, and the complexities of the tumor microenvironment continue to limit treatment efficacy (2–4). The discovery and validation of cancer biomarkers are essential for the early diagnosis of cancer, monitoring treatment response, and evaluating prognosis. However, many cancer biomarkers suffer from insufficient specificity and sensitivity, which limits their accuracy in early diagnosis and efficacy monitoring (5, 6). In light of the increasing global cancer burden, identifying new cancer biomarkers is crucial for enhancing treatment efficacy and improving patient prognosis.

The transmembrane emp24 domain-containing protein (TMED) family comprises four subfamilies: the α subfamily (TMED4 and TMED9), β subfamily (TMED2), γ subfamily (TMED1, TMED3, TMED5, TMED6, and TMED7), and δ subfamily (TMED10), totaling nine members (7). These proteins are key regulators of protein transport in the human body and play significant roles in the development of various diseases, including malignant tumors. Among them, TMED9 has garnered attention due to its essential role in numerous biological processes. Recent studies indicate that TMED9 overexpression in ovarian cancer correlates with poor overall survival, while in breast cancer, high TMED9 expression is associated with enhanced tumor proliferation and migratory capacity (8, 9). The role of TMED9 in hepatocellular carcinoma is also increasingly recognized, as its expression levels correlate with tumor vascular invasion and unfavorable prognosis (10). Additionally, research has demonstrated that TMED9 promotes colon cancer metastasis by activating CNIH4/TGFα/GLI signaling (11). Although TMED9’s role in specific cancer types has been investigated, there is a lack of a comprehensive perspective to evaluate the expression patterns and functions of TMED9 across different cancer types. This limitation restricts our in-depth understanding of TMED9’s mechanisms of action in the context of pan-cancer.

In our study, we analyzed the expression pattern, diagnostic value, prognostic significance, copy number variations, and epigenetic changes of TMED9 in pan-cancer using multi-omics data. Specifically, we focused on the immune characteristics and functional role of TMED9 in glioma, as well as its response to immunotherapy. Additionally, we investigated the correlation between TMED9 and the metastasis, migration, and invasion of glioma cells through cellular experiments. This study aims to provide a comprehensive analysis of the role of TMED9 in various cancers and its impact on the clinical significance and prognostic value of glioma. Furthermore, we seek to elucidate its functional mechanisms and offer new insights and strategies for precision medicine and the comprehensive treatment of glioma.

## 2 Materials and method

### 2.1 Datasets acquisition

The raw RNA-seq and clinical data are available at the Pancancer Atlas publication page (https://gdc.cancer.gov/about-data/publications/pancanatlas). To increase the number of normal samples, the normal sample TPM expression data from the Genotype-Tissue Expression (GTEx) project were paired with tumor TPM expression data from The Cancer Genome Atlas (TCGA). To ensure accuracy and eliminate the influence of anatomical factors, only primary tumor tissues from TCGA were retained for pairing with the GTEx data. The data were standardized into unitless Z-scores using the formula (x-μ)/σ. The pan-cancer transcript expression profiles and copy number variation data were obtained from the UCSC XENA website (https://xenabrowser.net/datapages/). The pan-cancer expression quantitative trait loci Genome-Wide Association Study (eQTL-GWAS) co-localization analysis data were sourced from the Open GWAS website (https://gwas.mrcieu.ac.uk/) (12). The pan-cancer immune cell infiltration data were downloaded from the Tumor Immune Estimation Resource 2.0 (TIMER2.0, http://timer.cistrome.org/) (13). The protein expression data from the reverse phase protein array were retrieved from The Cancer Proteome Atlas (TCPA, https://tcpaportal.org/index.html) database. The pan-cancer immune subtype data were obtained from prior studies (14). The immune inflammation-related gene set was downloaded from the official website of the Kyoto Encyclopedia of Genes and Genomes (KEGG, https://www.kegg.jp/). The scRNA-seq data were acquired from Tumor Immune Single-cell Hub 2 (TISCH2, http://tisch.comp-genomics.org/) (15). The spatial transcriptome data were sourced from previous studies (16). The Cancer-Immunity Cycle data were retrieved from the Tracking Tumor Immunophenotype (TIP, http://biocc.hrbmu.edu.cn/TIP/) database (17). Lastly, the RNAss tumor stemness score for glioma was drawn from earlier research (18). The abbreviations for all cancers can be found in **Supplementary Table 1**.

The validation dataset for gliomas comes from the Chinese Glioma Genome Atlas (CGGA, http://www.cgga.org.cn/), Gene Expression Omnibus (GEO, https://www.ncbi.nlm.nih.gov/geo/), and ArrayExpress databases (https://www.ebi.ac.uk/biostudies/arrayexpress) (19). They are CGGA-301, CGGA-325, CGGA-693, GSE16011 (20), GSE61335 (GPL19184) (21), GSE33331 (22), GSE42669 (23), GSE72951 (24), E-MTAB-3892 (25), and E-TABM-898 (26), which are used for the expression analysis, survival analysis, and functional enrichment analysis of TMED9. Additionally, datasets GSE100736, GSE138863 (27), and GSE138942 (28) from GEO are used to assess the expression differences of TMED9 under different drug treatments. The probe matrix was annotated as a gene matrix based on the platform file for each dataset. When a single gene corresponded to multiple probes, the gene expression value was determined by averaging the expression levels of those probes. Subsequently, the data were normalized to unitless Z-score values using the formula (x−μ)/σ.

### 2.2 Expression landscape and genomic alterations of TMED9 in pan-cancer

The differential expression of TMED9 in human normal tissues was initially analyzed using the GTEx database. Subsequently, the expression differences of TMED9 across various immune cells and tumor cell lines were assessed using the Human Protein Atlas (HPA) database. Additionally, a pan-cancer expression profile was employed to examine the differential expression of TMED9 between normal and tumor tissues. The gganatogram package was utilized to create organ diagrams that visualize the median z-scores of tumor and normal groups for each organ. Furthermore, the Gene Expression database of Normal and Tumor tissues 2 (GENT2, http://gent2.appex.kr/gent2/) was used to further confirm the differential expression of TMED9 between tumor and normal tissues (29). Finally, the Proteomics module in the UALCAN database facilitated the analysis of differential protein expression of TMED9 in both tumor and normal tissues (30), while immunohistochemistry results from the HPA database were utilized to further corroborate the differential protein expression of TMED9.

The cBioPortal for Cancer Genomics was utilized to analyze the frequencies of genomic mutations, amplifications, and deep deletions in pan-cancer studies. Additionally, we focused on the copy number variation of TMED9 across various cancer types. The methylation changes of TMED9 were assessed using the Shiny Methylation Analysis Resource Tool (SMART, http://www.bioinfo-zs.com/smartapp/) (31). Kaplan-Meier curves were generated using the Tumor Immune Dysfunction and Exclusion (TIDE, http://tide.dfci.harvard.edu/) methylation module to evaluate the prognostic relevance of TMED9 promoter methylation (32). The ssGSEA method from the GSVA package was employed to compute scores for different mutation-related gene sets in pan-cancer, and Spearman correlation analysis was conducted to examine the relationship between the scores of these gene sets and TMED9 expression (33). Finally, pan-cancer eQTL-GWAS colocalization analysis was carried out with the coloc package using default parameters, where the cutoff for colocalization evidence was defined as PP.H4.abf greater than 0.75, followed by visualization with the stack_assoc_plot function in the gassocplot2 package.

### 2.3 Immune correlation analysis

We collected 150 genes associated with immune regulators from the study by Charoentong et al., which included 41 chemokines, 18 receptors, 21 major histocompatibility complex (MHC) molecules, 24 immunosuppressive factors, and 46 immunostimulatory factors (34). The Pearson correlation coefficient was employed to assess the relationship between these genes and TMED9 in pan-cancer. Based on the median expression value of TMED9, tumor samples were categorized into high-expression and low-expression groups, and the proportion of each subtype within these groups was calculated; significance was determined using the chi-square test. The TIMER2.0 database was utilized to investigate the abundance of different cell types within the tumor microenvironment across 33 cancer types. The ssGSEA method from the GSVA package was employed to calculate enrichment scores for immune-inflammation-related function sets, and the correlation between these scores and TMED9 gene expression was analyzed. The easier package was used to evaluate the cytolytic activity (CYT) score, tertiary lymphoid structure (TLS) score, interferon-γ (IFN-γ) score, T cell inflammation (T cell_inflamed) score, and chemokine score (35). Additionally, the TIDE algorithm was applied to predict potential responses to immunotherapy. Patients with high TIDE scores exhibited poor treatment efficacy and shorter survival following immune checkpoint blockade (ICB). Finally, the ROC Plotter server (https://rocplot.com/) and the Kaplan-Meier Plotter server (https://kmplot.com/analysis/) were employed to validate the association between TMED9 and immunotherapy (36, 37).

### 2.4 Functional enrichment analysis

The GeneMANIA server (http://genemania.org/) was utilized to predict genes that may interact with TMED9 (38). The ComPPI database (https://comppi.linkgroup.hu/) was employed to filter out interacting proteins that do not share common subcellular localization, allowing for the identification of proteins that interact with the TMED9 gene (39). In the pan-cancer samples, the top 30% of samples exhibiting the highest TMED9 expression were classified as the high expression group, while the bottom 30% were classified as the low expression group. Differential analysis was performed using the limma package, and the log2FC values for each gene were calculated and sorted. Subsequently, the gene set enrichment analysis (GSEA) function from the clusterProfiler package was applied (40). Based on protein expression data from the TCPA database and published research findings, pathway activity scores for 10 cancer-related pathways (TSC/mTOR, RTK, RAS-MAPK, PI3K-AKT, ER hormone, AR hormone, EMT, DNA damage response, cell cycle, and apoptosis) were computed, and statistical analyses were conducted to compare pathway activity scores between the high and low expression groups of TMED9 (41). The CancerSEA database integrates characteristic gene expression profiles for 14 tumor cell states (42). The z-score algorithm from the R package GSVA was utilized to calculate the activity of each pathway and to obtain a combined z-score (43). Subsequently, the scores were standardized using the scale function, defined as the gene set score, and the Pearson correlation between the TMED9 gene and the gene set score was calculated.

### 2.5 Evaluation of the diagnostic and prognostic value of TMED9

Receiver operating characteristic (ROC) analysis was conducted using the pROC package, calculating the 95% confidence intervals and area under the curve (AUC) values, while also generating ROC curves to evaluate the diagnostic efficacy of TMED9 gene expression in differentiating between tumor and normal groups. Univariate Cox regression and Kaplan-Meier survival analyses were performed using the survival package in R, where the hazard ratio (HR) and 95% confidence interval (CI) were employed to quantify relative risk. The cutoff values for high and low expression groups were established with the survminer package, ensuring that each group comprised at least 30% of the total sample to mitigate over-grouping bias. The log-rank test, executed by the survfit function, assessed the significance of survival differences between the two groups. Based on TMED9 gene expression, patients were stratified into four groups: Q1 (highest 25% expression), Q2, Q3, and Q4 (lowest 25% expression). A chi-square test was applied to determine the significance of differences in patient composition among these groups. The survival package was also utilized for multivariate Cox survival analysis of both TMED9 gene and clinical variables, evaluating the potential of TMED9 as an independent prognostic factor.

### 2.6 Single-cell and spatial transcriptomic analysis

Gene expression files at single-cell resolution in glioma were obtained from the TISCH2 database, and the pheatmap package was employed to construct a heatmap visualizing the single-cell expression landscape of the TMED9 gene in glioma. The Uniform Manifold Approximation and Projection (UMAP) method was utilized for dimensionality reduction of the gene expression data. Specifically, the Nebulosa package was employed to estimate weighted kernel density for enhanced visualization of the single-cell data (44). Building on previous studies (45), the Cottrazm package facilitated the deconvolution of cell composition in spatial transcriptome slices (46), calculating the predominant cell type in each microregion, and visualizing the results using the SpatialPlot function from the Seurat package. The scale function performed z-score normalization, and the pheatmap package was used for heatmap visualization, enabling observation of the average expression of the TMED9 gene across different cell types in each slice. The SpatialFeaturePlot function of the Seurat package visualized the expression landscape of the TMED9 gene in each microregion. Additionally, Spearman correlation analysis assessed the relationships between cell content across all spots and the correlation between cell content and gene expression, with results visualized using the linkET package.

### 2.7 Analysis of tumor stemness and drug sensitivity

We assessed the correlation between TMED9 and tumor stemness in glioma by utilizing RNAss tumor stemness scores and mRNAsi derived from the one-class logistic regression (OCLR) algorithm (18). For drug sensitivity analysis, we selected 198 drugs from the Genomics of Drug Sensitivity in Cancer (GDSC) database. Specifically, the R package oncoPredict was employed to predict drug sensitivity based on TMED9 gene expression (47). Using Spearman correlation analysis, we calculated the relationship between TMED9 gene expression and the half-inhibitory concentration (IC50) of antagonists measured in the GDSC database. A negative correlation suggests that increased gene expression is associated with heightened sensitivity to the drug, while a positive correlation indicates that increased gene expression correlates with enhanced resistance to the drug. Furthermore, to explore potential therapeutic options to mitigate the tumor-promoting effects mediated by the TMED9 gene, we conducted Connectivity Map (cMAP) analysis. In the context of Pan-Cancer, TMED9 gene-related features were compared with cMAP gene features using the optimal feature matching method XSum (eXtreme Sum) through the cMAP database, yielding similarity scores for 1,288 compounds (48). Compounds with low similarity scores may inhibit gene-mediated cancer-promoting effects.

### 2.8 Cell culture and Quantitative real-time PCR

The U-87 MG and U251 cell lines were obtained from Procell Life Science & Technology (Wuhan, China). The U251 cells were cultured in DMEM medium (HyClone, USA) supplemented with 10% fetal bovine serum (Vazyme, China), 100 mg/mL streptomycin, and 100 U/mL penicillin. In contrast, the U-87 MG cells were cultured in MEM medium (Gibco, USA), also supplemented with 10% fetal bovine serum (Vazyme, China), 100 mg/mL streptomycin, and 100 U/mL penicillin. All cells were incubated in a humidified atmosphere containing 5% CO2 at 37°C.

Total RNA was isolated using TRIzol reagent (Invitrogen, USA) according to the manufacturer’s instructions. One microgram (1 µg) of RNA was utilized for complementary DNA (cDNA) synthesis employing the Hiscript III First Strand cDNA Synthesis Kit (Vazyme, China). GAPDH was amplified from each sample to ensure equal cDNA input. Each polymerase chain reaction (PCR) contained 1 µL of cDNA, 0.6 µL of forward and reverse primers (10 µM), 7.5 µL of ChamQ Universal SYBR qPCR Master Mix (Vazyme, China), and 6.3 µL of double-distilled water (ddH2O). The PCR parameters consisted of an initial denaturation step at 95°C for 10 minutes, followed by 40 cycles of denaturation at 95°C for 15 seconds, annealing at 62°C for 1 minute, and extension at 72°C for 15 seconds. A final extension phase included one reaction at 60°C for 1 minute and an additional step at 95°C for 15 seconds. The forward and reverse primers for GAPDH were GGAGCGAGATCCCTCCAAAAT and GGCTGTTGTCATACTTCTCATGG, respectively, while the primers for TMED9 were GCGCTCTACTTTCACATCGG and CACCTCCACAAACATGCCAA,respectively.

### 2.9 Western blotting

Cells were harvested following treatment with small interfering RNAs (siRNAs). They were subsequently collected by centrifugation after being washed three times with phosphate-buffered saline (PBS). Protease inhibitors (Solarbio, China) were added to RIPA buffer to prepare total protein extracts. Antibodies for TMED9, GAPDH, MMP14, Vimentin, and MMP2 were obtained from Proteintech (China) and employed according to the manufacturer’s instructions for Western blot analysis. Goat Anti-Rabbit IgG-HRP (Proteintech, China) served as the secondary antibody. GAPDH was utilized as the loading control. Enhanced chemiluminescence (ECL) reagent (4A Biotech, China) was used to visualize the signals.

### 2.10 Transwell migration and invasion assay

The 24-well Transwell chamber (Corning Costar, USA) was prepared and stored at 4°C overnight. A total of 2 × 10^4 transfected U-87 MG and U251 cells were seeded in the upper chamber, with or without Matrigel, and incubated in serum-free medium, while the lower chamber was filled with 10% serum medium. After 48 hours, the Transwell chamber was removed, fixed with 4% paraformaldehyde for 15 minutes, and stained with crystal violet for 5 minutes. Finally, images were captured and observed using an optical microscope.

### 2.11 Statistical analysis

All data were processed using web tools and R software (version 4.0.3; https://www.r-project.org/). Pearson correlation analysis was conducted for normally distributed data, while Spearman correlation analysis was employed for non-normally distributed data. The Kruskal-Wallis rank sum test and Wilcoxon rank sum test were utilized to identify differences between multiple variables or two variables. The Kaplan-Meier method implemented the log-rank test to evaluate significance. Cell experimental data were analyzed with GraphPad Prism for Windows (version 9.0.0), and each experiment was repeated three times. Statistical significance was determined using Student’s t-test, with p values less than 0.05 considered statistically significant. All statistical tests were two-tailed. The significance levels are indicated by the following symbols: *p < 0.05, **p < 0.01, ***p < 0.001.

## 3 Results

### 3.1 TMED9 is significantly upregulated in multiple tumor tissues

**Figure 1:**
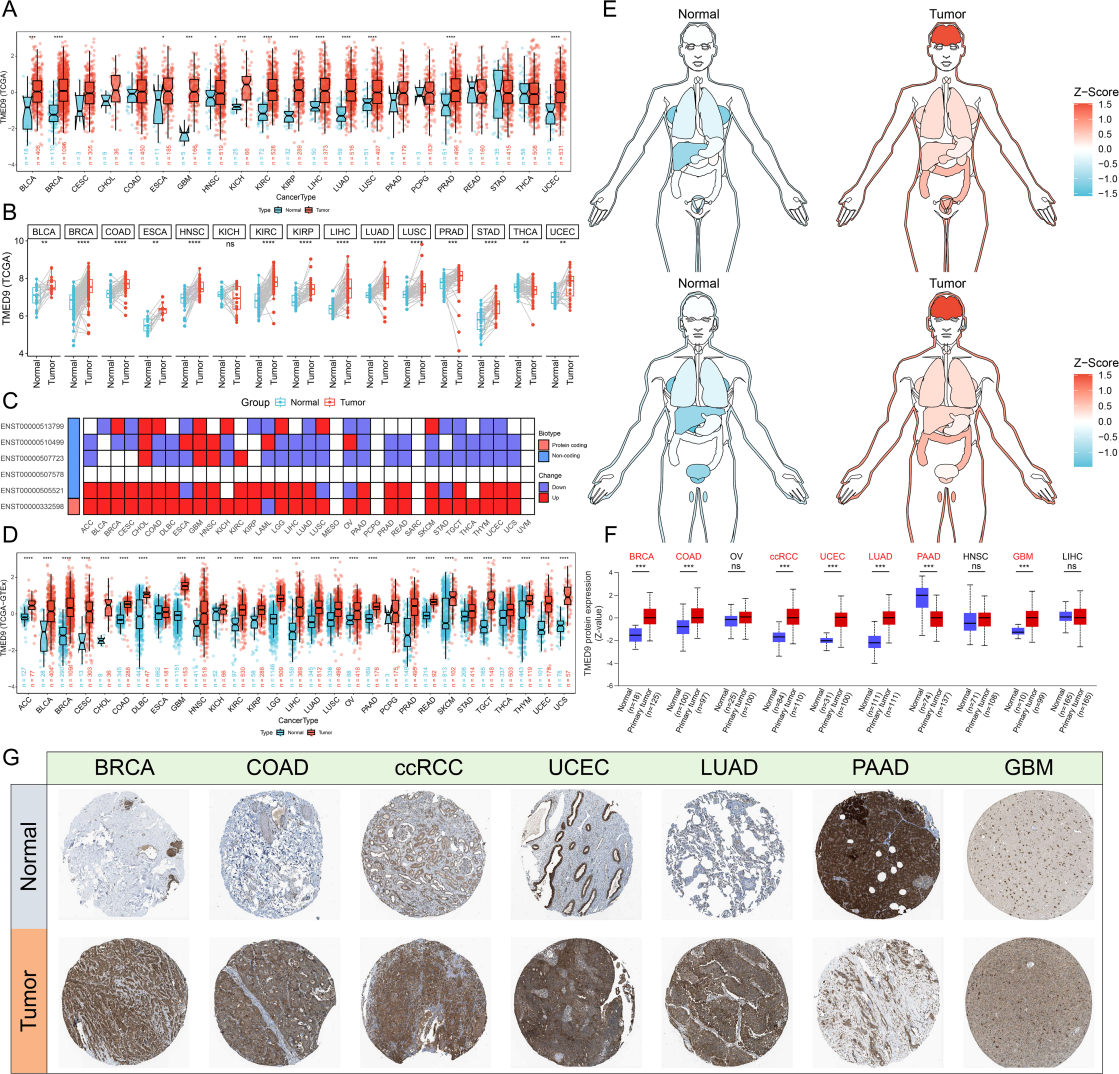
**(A)** Evaluation of differential expression of TMED9 in TCGA normal and tumor samples; **(B)** Evaluation of differential expression of TMED9 in tumor samples and paired normal samples in TCGA; **(C)** Evaluation of differential expression of TMED9 transcripts between normal and tumor samples in pan-cancer; **(D)** Evaluation of differential expression of TMED9 using TCGA dataset combined with GTEx normal samples; **(E)** Differential expression of TMED9 in different organ-related cancer and normal samples; **(F)** Differences in protein expression levels of TMED9 in pan-cancer; **(G)** Immunohistochemistry data validates the differential protein expression of TMED9 between different cancer tumor samples and normal samples (HPA datasets). *p < 0.05, **p < 0.01, ***p < 0.001, ****p < 0.0001, ns: not significant.

The analysis based on the human pan-normal tissue expression profile revealed that TMED9 is significantly overexpressed in tissues such as the pancreas, liver, and salivary glands (**Supplementary Figure 1A**). Immune cell analysis indicated that TMED9 is primarily expressed in dendritic cells and monocytes (**Supplementary Figure 1B**). Furthermore, high levels of TMED9 expression were observed in tumor cell lines, including kidney cancer, brain cancer, and myeloma (**Supplementary Figure 1C**). In the differential analysis of tumor and normal samples from the TCGA, we found that TMED9 is significantly overexpressed in more than half of the tumor types (**Figures 1A, B**). Transcript differential analysis demonstrated that, compared to normal tissues, the protein transcripts encoded by TMED9 exhibit high expression in nearly all tumor types (**Figure 1C**). Subsequent differential analysis utilizing normal samples from the GTEx database also revealed significant upregulation of TMED9 expression across nearly all cancer types (**Figures 1D, E**). Analysis from the GENT2 database further corroborated these findings (**Supplementary Figure 1D**). Data from the UALCAN database indicated that TMED9 protein levels were elevated in breast invasive carcinoma (BRCA), colon adenocarcinoma (COAD), clear cell renal cell carcinoma (ccRCC), uterine corpus endometrial carcinoma (UCEC), lung adenocarcinoma (LUAD), and glioblastoma multiforme (GBM), whereas a significant downregulation was observed in pancreatic adenocarcinoma (PAAD) (**Figure 1F**, **Supplementary Figure 1E**). Additionally, immunohistochemistry results from the HPA database confirmed the elevated expression of TMED9 in several tumors, including BRCA, COAD, and GBM (**Figure 1G**). We also assessed the correlation between TMED9 and clinical characteristics of tumors. The results demonstrated that in kidney renal clear cell carcinoma (KIRC), brain lower grade glioma (LGG), and liver hepatocellular carcinoma (LIHC), TMED9 expression progressively increased with tumor progression. Notably, we observed significantly high TMED9 expression during the M1 stage of head and neck squamous cell carcinoma (HNSC), KIRC, and mesothelioma (MESO), while significantly low expression was evident in the M1 stage of LUAD, suggesting a potential association with tumor metastasis. Additionally, TMED9 was significantly associated with multiple tumor subtypes (**Supplementary Figures 2A, B**).

### 3.2 Genetic alterations of TMED9 in pan-cancer

**Figure 2:**
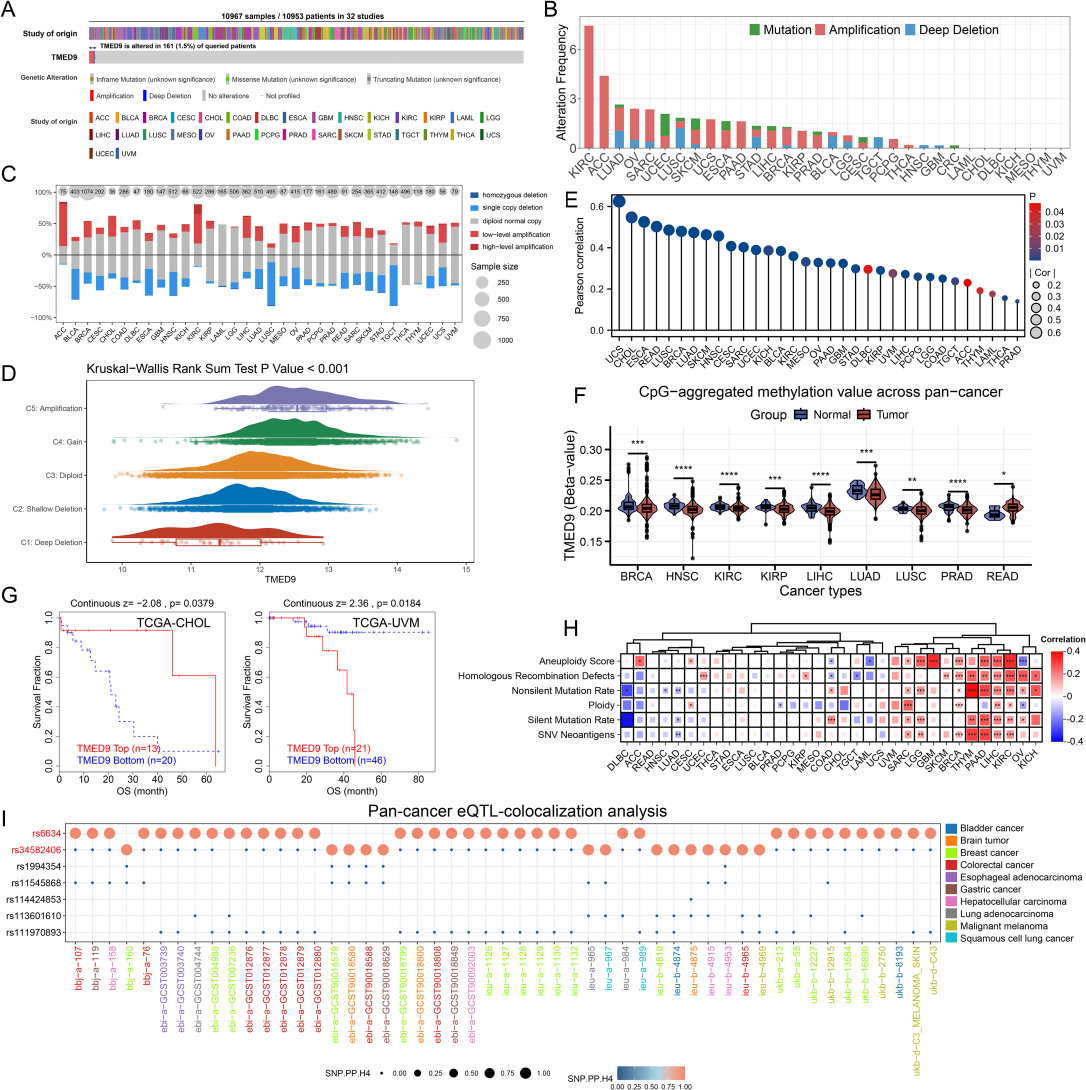
**(A)** Evaluation of the pan-cancer mutational landscape of TMED9 based on the cBioPortal database; **(B)** Mutation of TMED9 in different cancer types; **(C)** Copy number variation levels of TMED9 in pan-cancer; **(D)** TMED9 expression varies among different CNV types in pan-cancer; **(E)** Correlation between copy number variation and mRNA levels of TMED9 in pan-cancer; **(F)** Evaluation of the differential methylation levels of TMED9 in pan-cancer tumor samples and normal samples based on methylation data from the SMART database; **(G)** Association between TMED9 methylation and tumor prognosis; **(H)** Heat map of the correlation between TMED9 expression and different gene set scores in pan-cancer; **(I)** TMED9 co-localization results of eqTL-GWAS in pan-cancer. *p < 0.05, **p < 0.01, ***p < 0.001, ****p < 0.0001.

Analysis based on the cBioPortal server indicated that TMED9 experienced 161 mutations across 10,953 cancer patients, with KIRC exhibiting the highest mutation rate, predominantly in the form of amplification mutations (**Figures 2A, B**). Copy number variation analysis similarly revealed that TMED9 had a greater number of amplification mutations in various cancers, including adrenocortical carcinoma (ACC) and KIRC, while an increased incidence of deletion mutations was observed in bladder urothelial carcinoma (BLCA), lung squamous cell carcinoma (LUSC), and testicular germ cell tumors (TGCT) (**Figure 2C**). In pan-cancer analyses, the expression of TMED9 demonstrated an overall upward trend, transitioning from homozygous deletion to high copy number amplification (**Figure 2D**). Correlation analysis indicated that the copy number variation of TMED9 was significantly positively associated with its mRNA expression in almost all cancer types (**Figure 2E**). Methylation analysis revealed that, except for rectum adenocarcinoma (READ), TMED9 exhibited lower methylation levels in most tumors compared to normal tissues (**Figure 2F**). Furthermore, the methylation level of TMED9 was significantly correlated with the prognosis of cholangiocarcinoma (CHOL) and uveal melanoma (UVM) (**Figure 2G**). TMED9 was positively associated with multiple genomic scores in KIRC, LIHC, thymoma (THYM), PAAD, and LGG, suggesting that higher expression of TMED9 may correlate with increased chromosomal instability in these patients (**Figure 2H**). Additionally, eQTL-GWAS co-localization analysis demonstrated that rs6634 and rs34582406 had a PP.H4.abf value of 1 across all datasets, indicating that these variants of TMED9 may share genetic variations with multiple tumors (**Figure 2I**, **Supplementary Figures 3A–I**).

### 3.3 Immunological signature of TMED9 in pan-cancer

**Figure 3:**
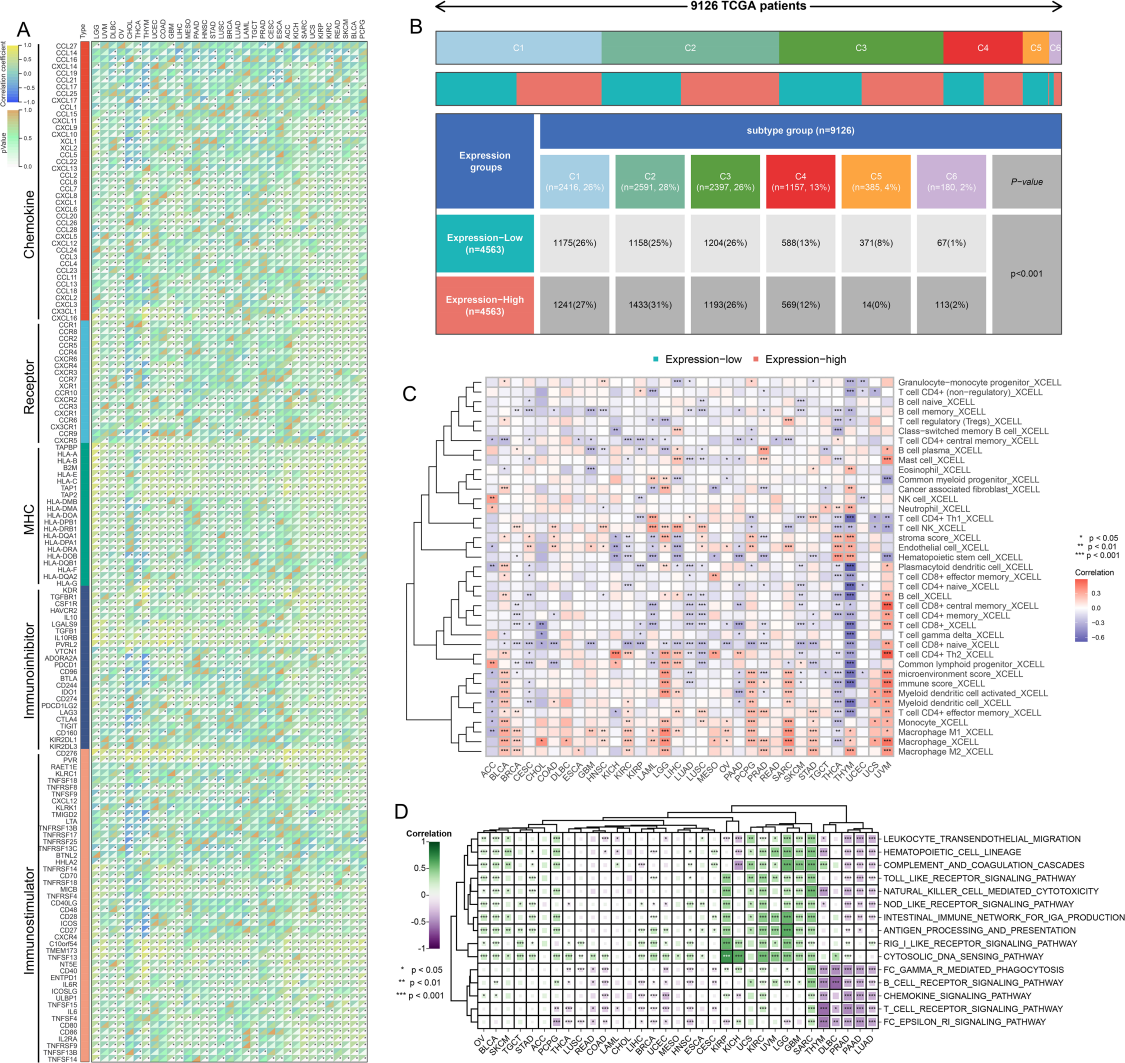
**(A)** Correlation between TMED9 and immune regulatory factors in pan-cancer; **(B)** Correlation between TMED9 and immune subtypes in pan-cancer; **(C)** Evaluation of the correlation between TMED9 and immune cell infiltration based on the xCELL algorithm; **(D)** Correlation between TMED9 and inflammation-related gene set scores in pan-cancer. *p < 0.05, **p < 0.01, ***p < 0.001.

The results of the correlation analysis demonstrated that TMED9 is positively correlated with various immune regulators, particularly MHC molecules. This finding indicates that the TMED9 high-expression group exhibits enhanced antigen presentation and processing capabilities (**Figure 3A**). Furthermore, multiple immunostimulators and immunoinhibitors show varying degrees of positive correlation with TMED9, thereby emphasizing the strong association between TMED9 and immunity. Immune subtype expression analysis revealed that, across pan-cancer, the C1 subtype (Wound Healing) and C2 subtype (IFN-γ Dominant) are predominant in the TMED9 high-expression group, a result confirmed across several specific cancer types (**Figure 3B**, **Supplementary Figure 4A**). Immune cell infiltration analysis indicated a significant positive correlation between TMED9 and various immune cell types, including macrophages, in BLCA, LGG, pheochromocytoma and paraganglioma (PCPG), sarcoma (SARC), THYM, and UVM (**Figure 3C**, **Supplementary Figures 4B–E**). Subsequently, we further validated the significant positive correlation between TMED9 and macrophages in LGG, SARC, THYM, and UVM using the TIMER2.0 online server (**Supplementary Figure 4F**). Additionally, we noted significant positive correlations between TMED9 and several inflammation-related scores across various tumors, including GBM, LGG, SARC, and KIRC (**Figure 3D**). These findings underscore the association between TMED9 and the tumor immune microenvironment.

### 3.4 TMED9 is significantly positively correlated with multiple tumor-related pathways

**Figure 4:**
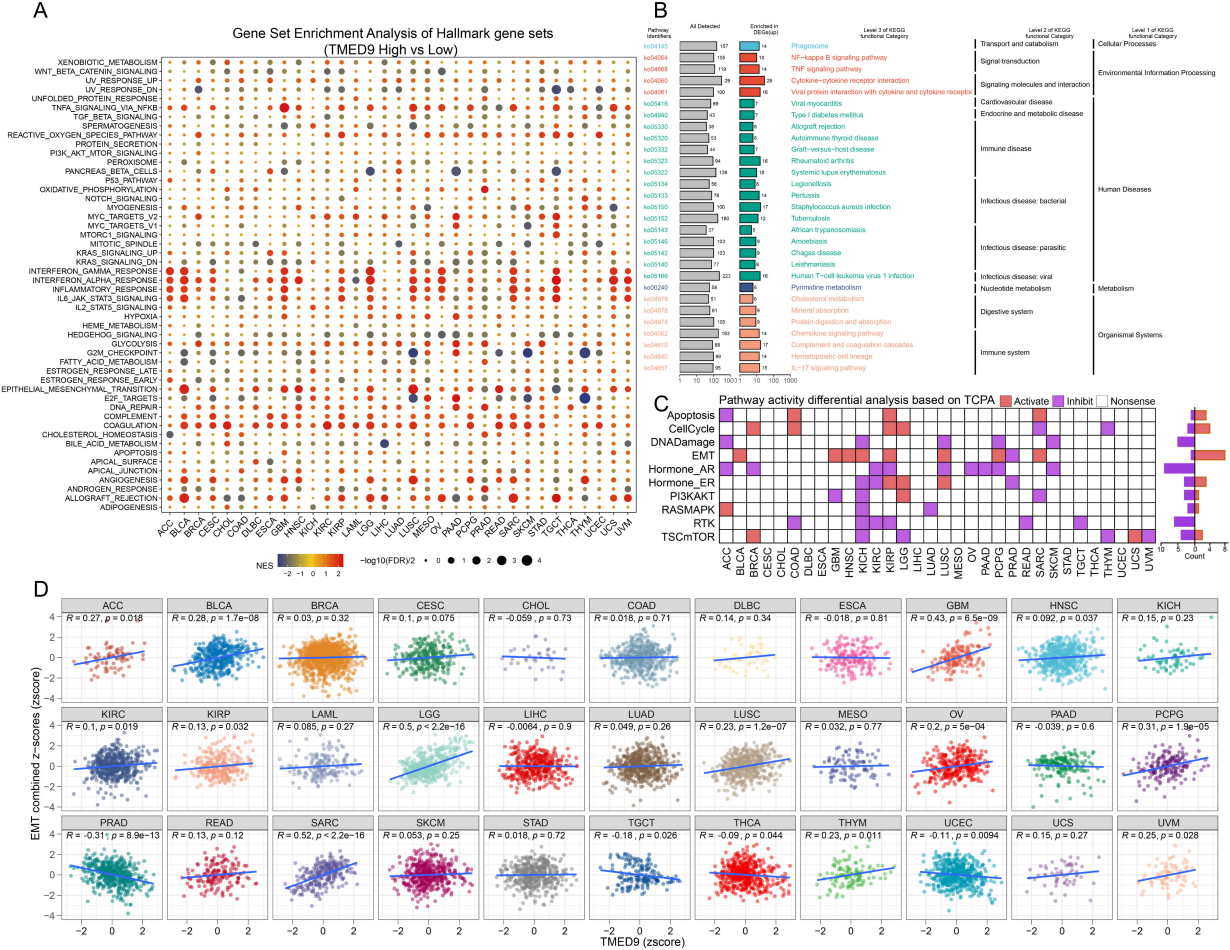
**(A)** Gene set enrichment analysis to evaluate the association between TMED9 and tumor-related pathways; **(B)** Pan-cancer KEGG functional enrichment analysis; **(C)** Evaluation of pathway activity differences between TMED9 high and low groups based on TCPA database; **(D)** Pearson correlation analysis between pan-cancer EMT signature score and TMED9.

Gene interaction network analysis revealed that TMED9 primarily interacts with TMED10, TMED2, and TMED1 (**Supplementary Figure 5A**). ComPPI facilitates the identification of proteins that may interact with TMED9 (**Supplementary Figure 5B**). Gene Set Enrichment Analysis (GSEA) results indicated that the TMED9 high-expression group is significantly enriched in immune-related pathways across cancers such as ACC, BLCA, GBM, LGG, and TGCT. Furthermore, TMED9 exhibits a significant positive correlation with TNF Signaling via NF-κB and Epithelial-Mesenchymal Transition (EMT) (**Figure 4A**). The KEGG enrichment analysis of highly expressed genes within the TMED9 high-expression group suggested that TMED9 is associated with various functions, particularly signaling molecules and their interactions, as well as immune and cancer-related pathways (**Figure 4B**). Additionally, proteomics-based pathway enrichment analysis demonstrated that the TMED9 high-expression group exhibits heightened activity in apoptosis, cell cycle, and EMT pathways across multiple tumors, with a particular emphasis on the EMT pathway (**Figure 4C**). The Gene Set Variation Analysis (GSVA) results revealed a significant positive correlation between TMED9 and the activities of EMT, invasion, and metastasis pathways in GBM, LGG, and SARC (**Figure 4D**, **Supplementary Figures 5C, D**).

### 3.5 TMED9 is an independent prognostic factor for glioma

**Figure 5:**
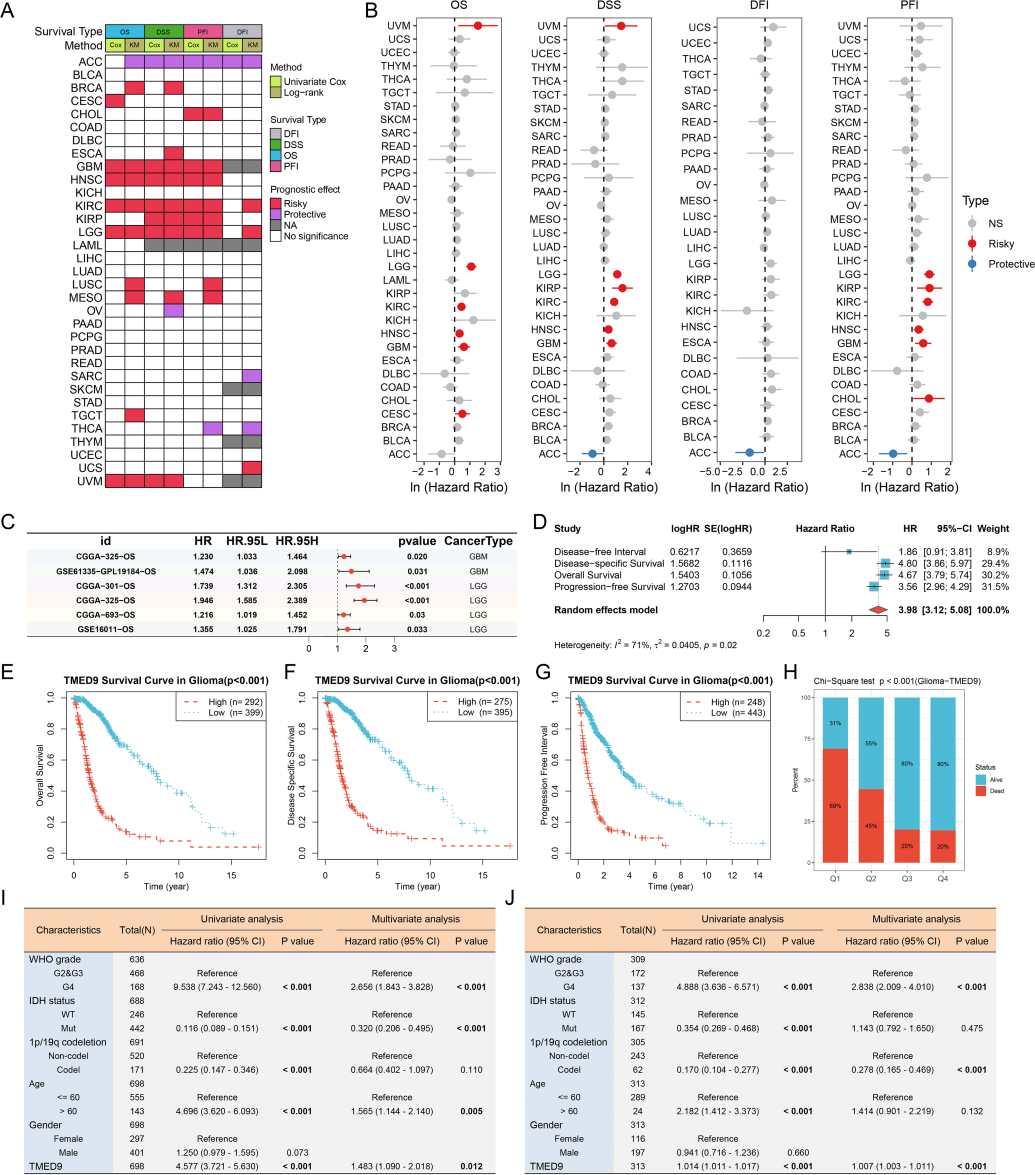
**(A)** Evaluation of the prognostic value of TMED9 in pan-cancer using univariate Cox analysis and KM survival analysis; **(B)** Univariate Cox survival analysis of four survival categories (OS, DSS, PFI, and DFI); **(C)** Forest plot showing the univariate Cox analysis results of TMED9 in the external glioma dataset; **(D)** Univariate Cox survival analysis of four survival periods (OS, DSS, PFI and DFI) in glioma (GBMLGG); **(E–G)** Kaplan-Meier survival analysis of three survival periods (OS, DSS, and PFI) in glioma (GBMLGG); **(H)** Chi-square test to evaluate the number of survival and death samples between different TMED9 expression groups; **(I)** Evaluation of the potential of TMED9 as an independent prognostic factor for glioma based on TCGA dataset; **(J)** Evaluation of the potential of TMED9 as an independent prognostic factor for glioma based on CGGA dataset.

ROC curves indicated that TMED9 may serve as a diagnostic biomarker for specific cancers (**Supplementary Figure 6**). The correlation between TMED9 and overall survival (OS), disease-specific survival (DSS), progression-free interval (PFI), and disease-free interval (DFI) was evaluated across 33 tumor types in the TCGA database. A heatmap summarized the relationship between TMED9 and various survival intervals in all cancers, which was assessed using the Unicox and Kaplan-Meier methods (**Figure 5A**). Univariate Cox regression analysis demonstrated that high TMED9 expression was significantly associated with poor OS in cervical squamous cell carcinoma and endocervical adenocarcinoma (CESC), GBM, HNSC, KIRC, LGG, and UVM. Elevated TMED9 expression was also identified as a risk factor for poor DSS in GBM, HNSC, KIRC, kidney renal papillary cell carcinoma (KIRP), LGG, and UVM, while serving as a protective factor in ACC. For DFI, high TMED9 expression functioned as a protective factor in ACC. Regarding PFI, high TMED9 expression acted as a risk factor in CHOL, GBM, HNSC, KIRC, KIRP, and LGG, while remaining a protective factor in ACC (**Figure 5B**). Kaplan-Meier curves were utilized to evaluate these four prognostic outcomes (**Supplementary Figures 7A–T**, **Supplementary Figures 8A–Q**). Furthermore, the results of proteomics-based survival analysis revealed that high TMED9 expression posed a risk factor for BRCA, GBM, HNSC, KIRC, LUSC, and pancreatic ductal adenocarcinoma (PDAC) (**Supplementary Figures 9A–F**). Overall, these results suggest that elevated TMED9 expression is generally associated with poorer prognostic outcomes in patients with GBM, LGG, HNSC, and KIRC.

**Figure 6:**
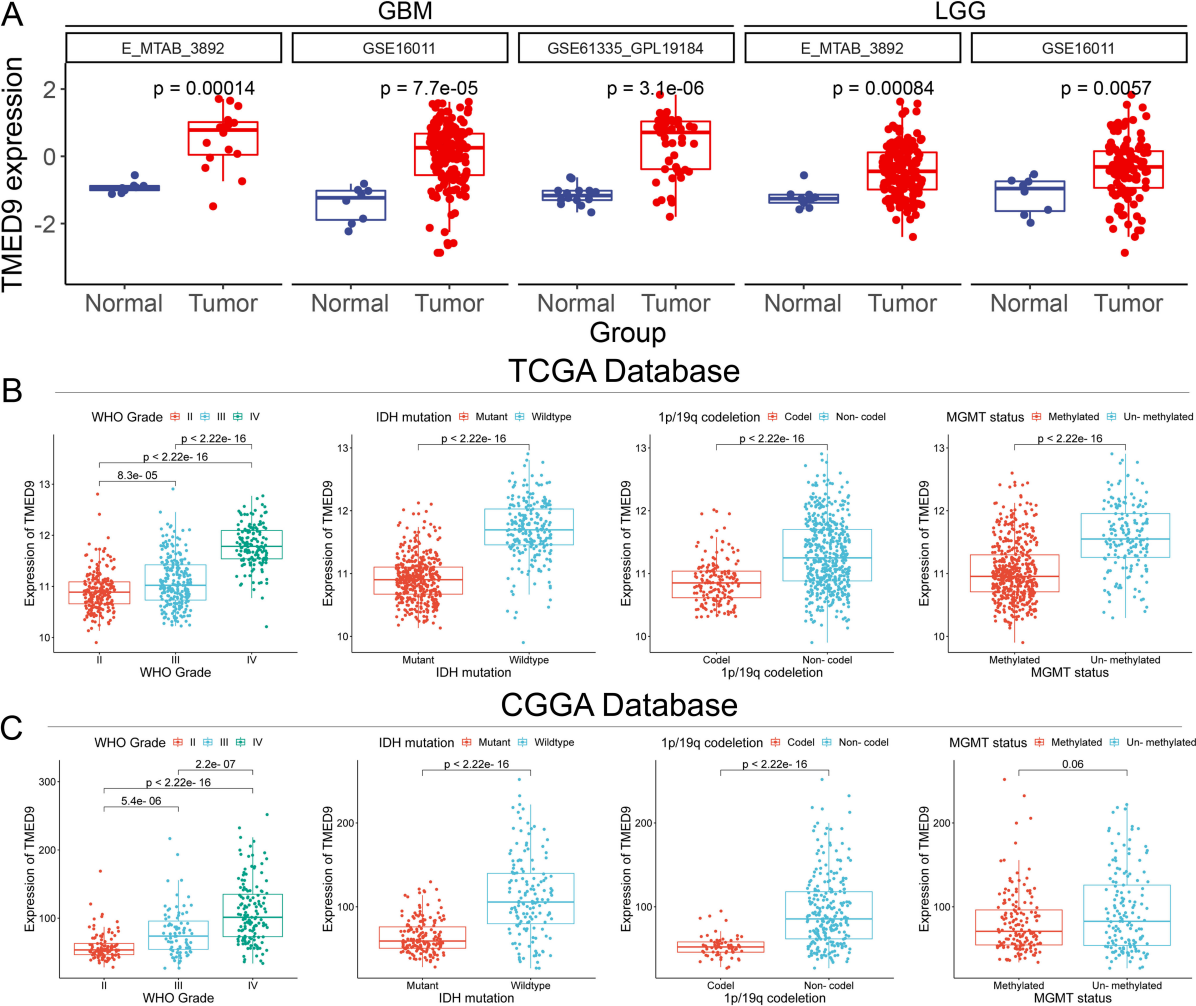
**(A)** Additional datasets validated the significant high expression of TMED9 in glioma tumor samples; **(B)** Evaluation of the association between TMED9 and clinical traits (Grade, IDH mutation, 1p/19q codeletion, and MGMT status) based on the TCGA dataset; **(C)** Evaluation of the association between TMED9 and clinical traits (Grade, IDH mutation, 1p/19q codeletion, and MGMT status) based on the CGGA dataset.

We concentrated on the clinical prognostic relationship between TMED9 and glioma. In several additional glioma datasets, we observed a significant association between high TMED9 expression and poor prognosis (**Figure 5C**, **Supplementary Figures 10A–F**). After merging expression profiles and clinical data from GBM and LGG, we found that high TMED9 expression correlated significantly with shorter OS, DSS, and PFI in glioma patients (**Figures 5D–G**). Moreover, we noted an elevated mortality rate in the top 25% of patients with the highest TMED9 expression levels (**Figure 5H**). Additionally, multivariate Cox analysis based on TCGA and CGGA datasets indicated that TMED9 is an independent prognostic factor for glioma (**Figures 5I, J**). Expression profile data from multiple datasets confirmed the significant upregulation of TMED9 in glioma (**Figure 6A**). Clinical correlation analysis revealed that TMED9 was highly enriched in high-grade gliomas and IDH wild-type gliomas within the TCGA database. Furthermore, samples lacking 1p/19q co-deletion exhibited higher TMED9 expression. The aforementioned results were corroborated in the CGGA database. In the TCGA database, TMED9 was highly expressed in samples without MGMT promoter methylation. The expression of TMED9 showed a similar trend in the CGGA database, although this difference was not statistically significant (**Figures 6B, C**). Overall, these findings indicate that TMED9 is significantly overexpressed in gliomas with greater malignancy.

### 3.6 TMED9 is significantly associated with glioma macrophages and immunotherapy

**Figure 7:**
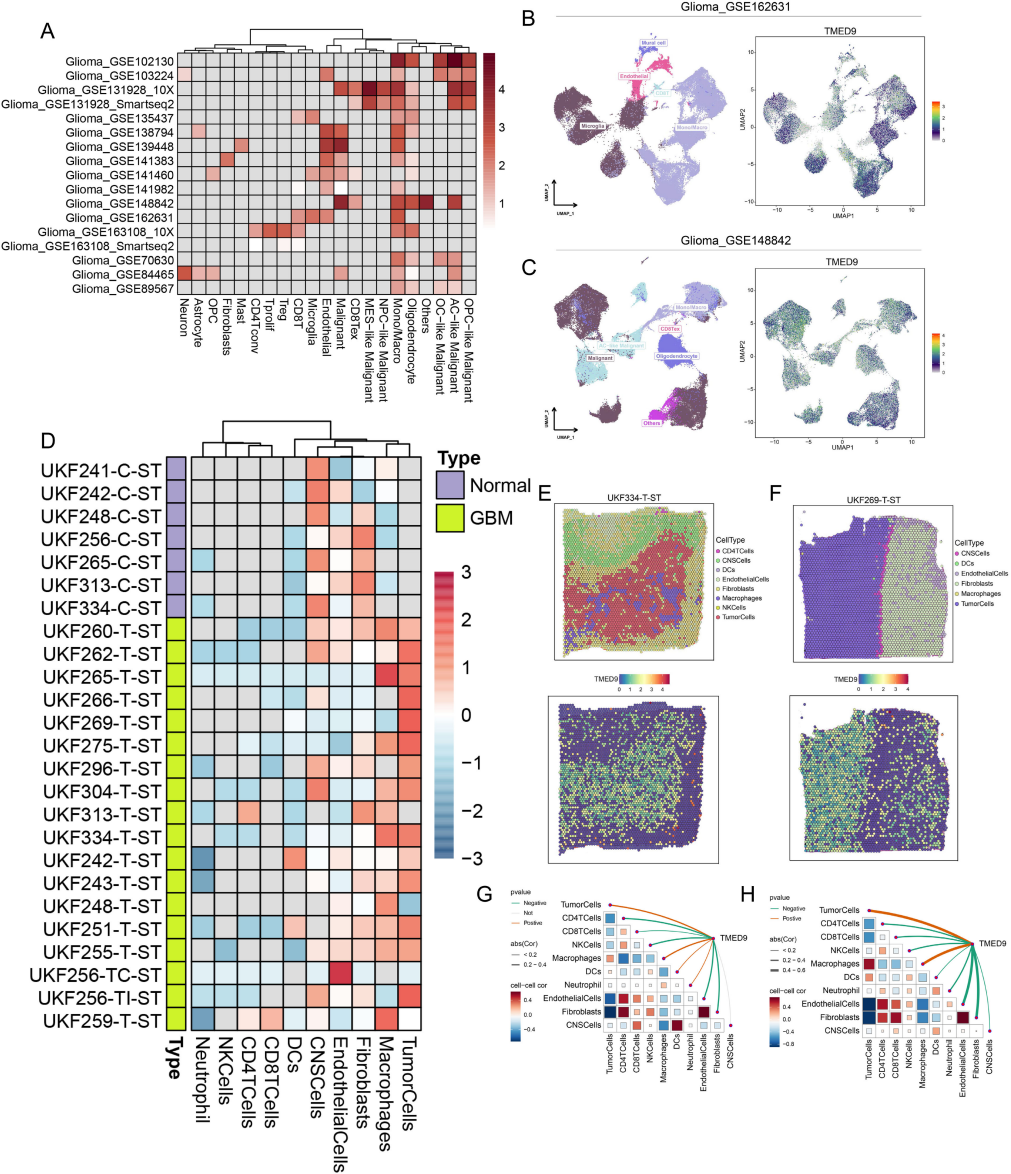
**(A)** Single-cell analysis evaluates the expression level of TMED9 in different cells in glioma; **(B, C)** TMED9 was observed to be significantly overexpressed in malignant cells and macrophages in the Glioma_GSE162631 and Glioma_GSE148842 datasets; **(D)** Expression of TMED9 gene in each microdomain in spatial transcriptome sections of glioma; **(E, F)** Expression localization of TMED9 gene in spatial transcriptome sections UKF334-T-ST and UKF269-T-ST; **(G, H)** Spearman correlation of TMED9 gene expression with each cell type in microdomains at idle resolution.

Single-cell analysis revealed that TMED9 is predominantly expressed in the malignant cells and monocytes/macrophages of gliomas (**Figure 7A**). The UMAP visualization and expression analysis of the Glioma_GSE162631 and Glioma_GSE148842 datasets confirmed the significant high expression of TMED9 in both malignant cells and macrophages (**Figures 7B, C**). Spatial transcriptome analysis indicated that in multiple normal glioma sections, TMED9 was primarily expressed in CNS cells and fibroblast microregions. Conversely, in various glioma sections, TMED9 showed dominant expression in tumor cells and macrophage microregions (**Figure 7D**). Single-gene localization analysis revealed that TMED9 expression is similar in tumor cells and macrophages, suggesting that within the context of glioma, TMED9 may primarily be expressed by these cell types (**Figures 7E, F**). Correlation analysis further supported these localization findings, demonstrating a significant positive correlation between TMED9 expression levels and the abundance of tumor cells and macrophages in the sampled regions (**Figures 7G, H**).

**Figure 8:**
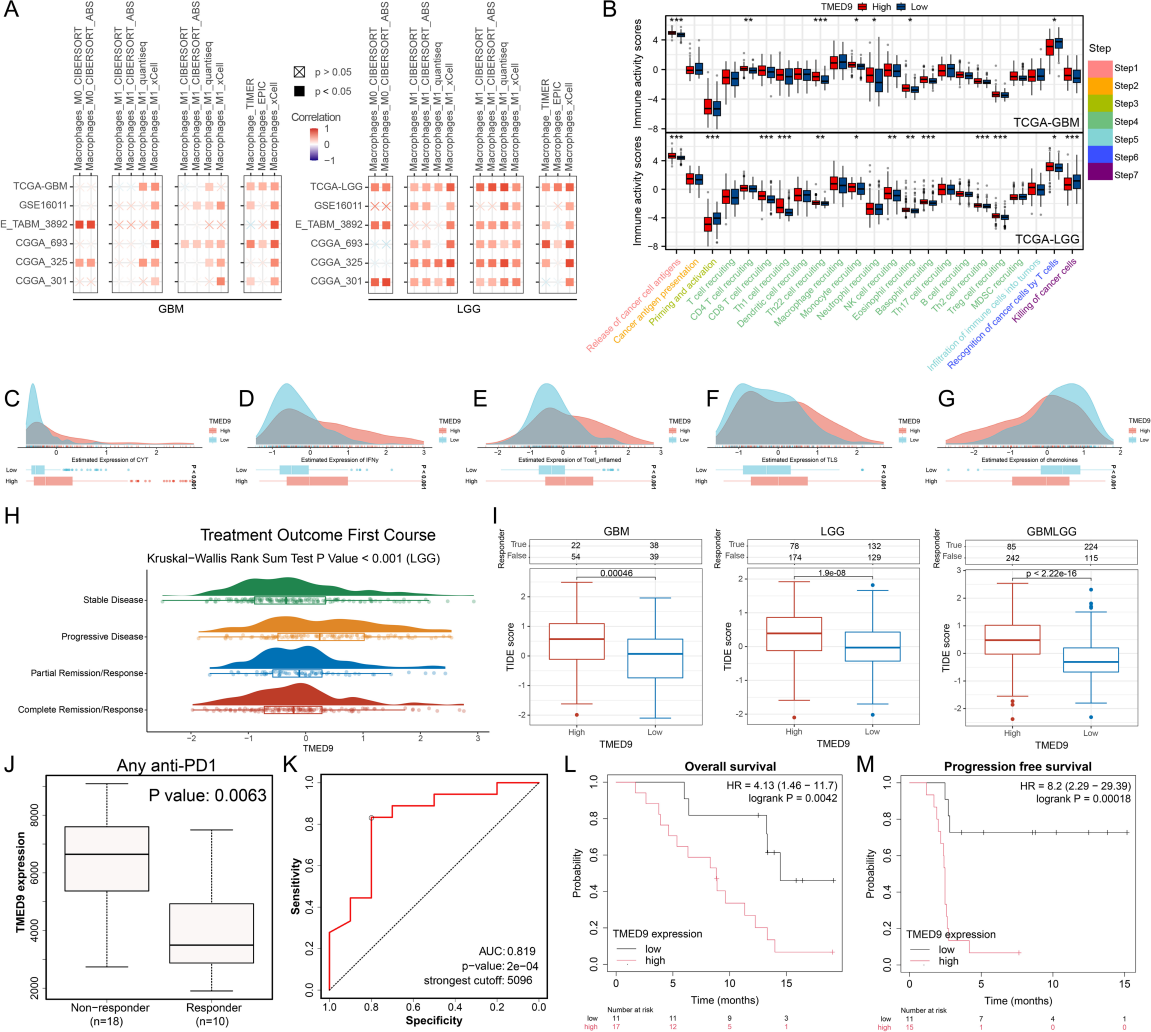
**(A)** Multi-algorithm calculation of Spearman correlation between TMED9 expression and immune infiltrating cells in glioma; **(B)** Evaluation of TIP score differences between high and low TMED9 expression groups in glioma; **(C-G)** Differences in CYT score, IFNy score, T cell_inflamed score, TLS score, and Chemokines score between high and low expression groups of TMED9 gene; **(H)** Difference in TMED9 expression in the first course of treatment outcomes of LGG; **(I)** TIDE algorithm evaluates the correlation between TMED9 and glioma immunotherapy; **(J)** TMED9 expression was lower in the immunotherapy response group; **(K)** ROC curve evaluation of TMED9’s predictive performance for immunotherapy efficacy; **(L, M)** The group with low TMED9 expression in glioma has longer overall survival and progression free survival when receiving immunotherapy. *p < 0.05, **p < 0.01, ***p < 0.001.

The analysis of immune cell infiltration across multiple glioma datasets revealed that TMED9 exhibited a significant positive correlation with macrophage infiltration, particularly pronounced in LGG, as illustrated in **Figure 8A**. The activity of the cancer immune cycle serves as a direct reflection of immune function. In GBM, we observed that both cancer cell antigen release (step 1) and immune cell trafficking to tumors (step 4)—including the recruitment of CD4 T cells, Th22 cells, monocytes, neutrophils, and eosinophils—were significantly upregulated in the high TMED9 expression group. Conversely, T cell recognition of cancer cells (step 6) was downregulated in this group. In LGG, a more pronounced correlation between TMED9 and the cancer immune cycle was observed. Specifically, within the high TMED9 group, cancer cell antigen release (step 1), immune cell trafficking (step 4)—which includes recruitment of CD8 T cells, Th1 cells, Th22 cells, monocytes, natural killer (NK) cells, eosinophils, basophils, Th2 cells, and regulatory T cells (Treg)—and T cell recognition of cancer cells (step 6) were upregulated, while priming and activation (step 3) and cancer cell killing (step 7) were downregulated, as shown in **Figure 8B**. Furthermore, higher CYT scores, IFNγ scores, T cell inflammation scores, and TLS scores, alongside lower chemokine scores, were observed in the high TMED9 group (**Figures 8C–G**). An evaluation of the relationship between TMED9 expression and glioma immunotherapy indicated that TMED9 was more highly expressed in the progressive disease group than in the partial or complete response groups (**Figure 8H**). Notably, the TMED9 high expression group exhibited a higher TIDE score, suggesting diminished efficacy of ICB and reduced survival following ICB treatment (**Figure 8I**). Additionally, we found that TMED9 expression in the immunotherapy response group markedly declined among patients receiving anti-PD1 treatment, with an AUC value of 0.819 for TMED9 in predicting anti-PD1 treatment outcomes (**Figures 8J, K**). Survival analysis revealed that, following immunotherapy, both OS and progression-free survival (PFS) were shorter in the high TMED9 expression group (**Figures 8L, M**).

### 3.7 Knockdown of TMED9 reduces glioma cell migration and invasion

**Figure 9:**
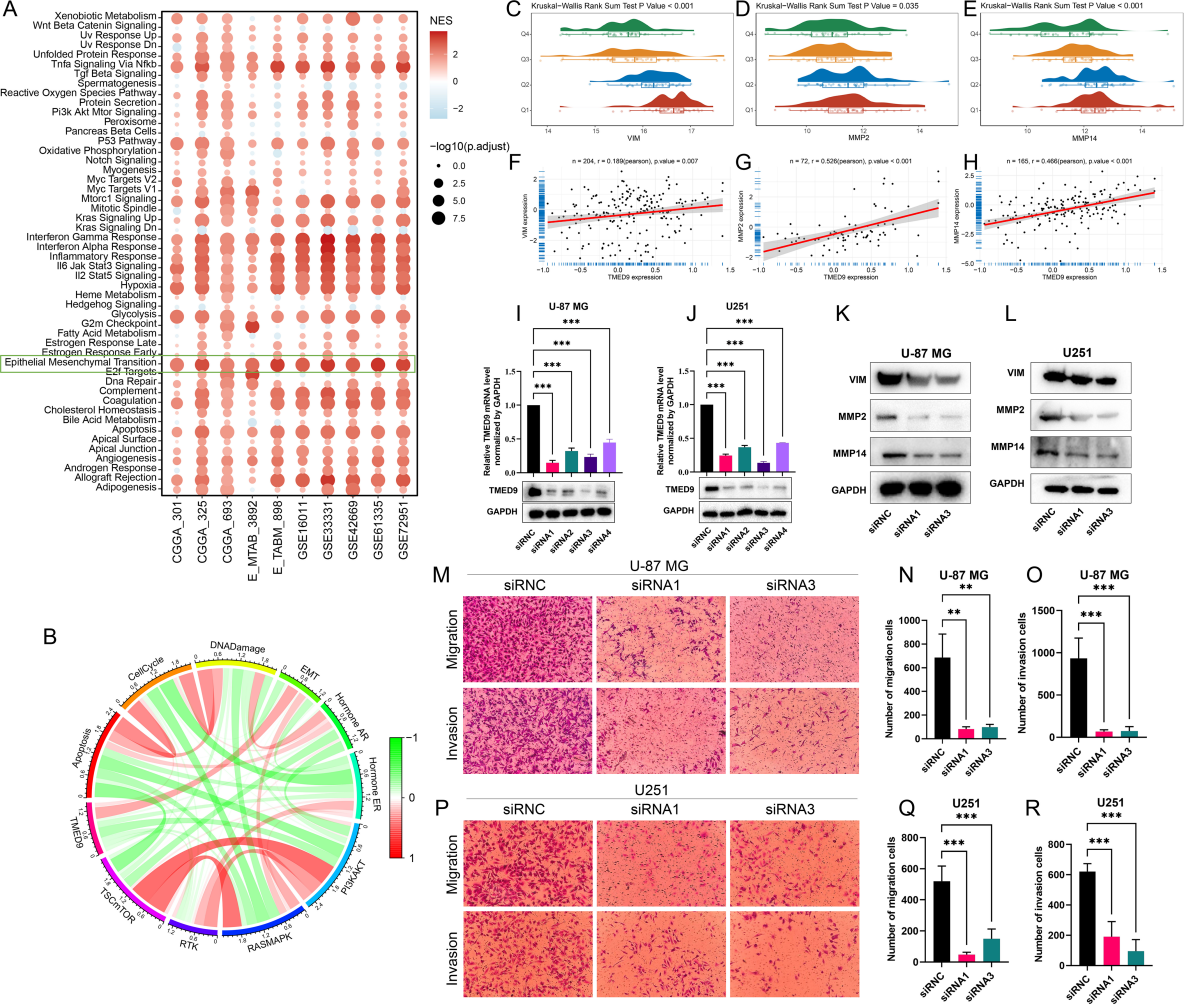
**(A)** Hallmark gene set enrichment analysis of multiple glioma datasets; **(B)** Correlation of TMED9 expression in gliomas with pathway-level quantification of functional proteins by TCPA-RPPA sequencing; **(C–E)** Evaluation of the correlation between TMED9 and EMT pathway-related genes VIM, MMP2, and MMP14 based on mRNA expression profiles; **(F–H)** Evaluation of the correlation between TMED9 and EMT pathway-related proteins VIM, MMP2, and MMP14 based on protein expression profile data; **(I, J)** Validation of TMED9 knockdown efficiency in U-87-MG and U251 cell lines using PCR and Western Blot assays; **(K, L)** Western Blot assay to evaluate the expression levels of EMT-related proteins after TMED9 knockdown in U-87-MG and U251 cell lines; **(M–O)** TMED9 knockdown reduces migration and invasion of the U-87-MG cell line; **(P–R)** TMED9 knockdown reduces migration and invasion of the U251 cell line. **p < 0.01, ***p < 0.001.

The GSEA analysis conducted on multiple glioma datasets revealed a significant positive correlation between TMED9 and the EMT pathway (**Figure 9A**; **Supplementary Figures 11A–J**). Additionally, pathway enrichment analysis of proteomics data further underscored this significant positive relationship (**Figure 9B**). Transcriptomic data analysis demonstrated that TMED9 was positively correlated with EMT-related genes, including VIM, MMP2, and MMP14 (**Figures 9C–E**). Correlation analysis using clinical proteomic tumor analysis consortium (CPTAC) proteomics data confirmed these findings (**Figures 9F–H**). To further investigate the relationship between TMED9 and the EMT pathway, we conducted cell experiments. In the glioma cell lines U-87MG and U251, we transfected cells with four distinct siRNAs to knock down TMED9 and performed RT-PCR and western blot analyses. The results indicated that both mRNA and protein expression levels of TMED9 in the transfected groups were significantly lower compared to the control group (**Figures 9I, J**). Subsequently, we selected siRNA1 and siRNA3, which demonstrated higher knockdown efficiency, for follow-up experiments. In the TMED9 knockdown group, significant reductions in the expression levels of VIM, MMP2, and MMP14 were observed (**Figures 9K, L**). Additionally, following TMED9 knockdown, we noted inhibited migration and invasion capabilities in U-87MG cells (**Figures 9M–O**), a finding that was corroborated in the U251 cell line (**Figures 9P–R**).

### 3.8 TMED9 is associated with glioma tumor stemness and drug sensitivity

**Figure 10:**
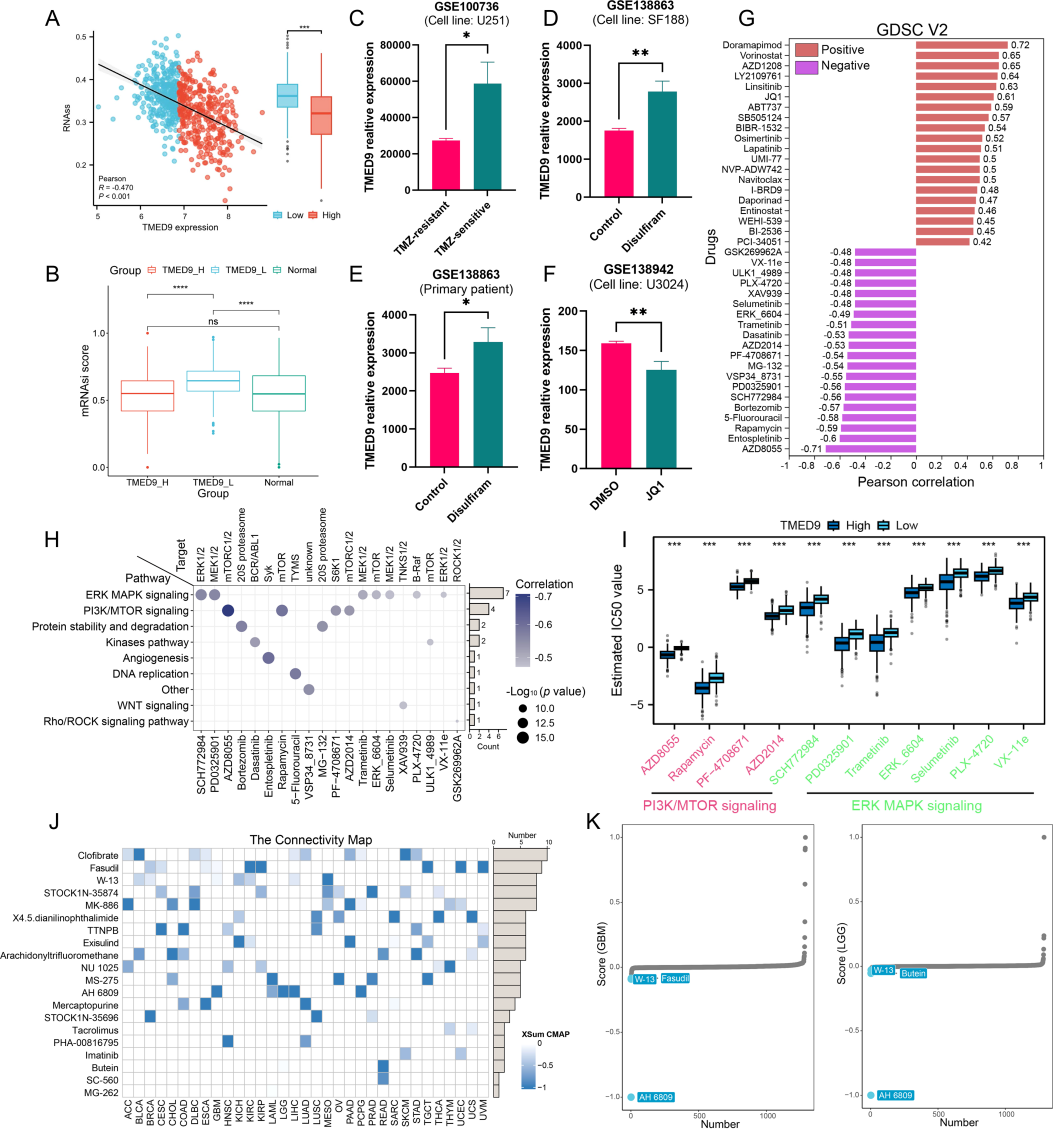
**(A)** Correlation between TMED9 and stemness score RNAss in glioma; **(B)** Correlation between TMED9 and stemness index mRNAsi in glioma; **(C–F)** Difference in the expression of TMED9 between drug-treated and control groups in glioma; **(G)** Spearman correlation of TMED9 with IC50 of drugs in the GDSC2 database. The correlation plot lists the top 20 drugs; **(H)** Target genes and pathways of the top 20 negatively correlated drugs; **(I)** Multiple drugs targeting the PI3K/MTOR signaling pathway and the ERK MAPK signaling pathway are significantly negatively correlated with TMED9; **(J)** In pan-cancer, the XSum algorithm is used to estimate potential small molecules and drugs that correct the biological effects caused by dysregulated TMED9 expression (from the cMap database); **(K)** In glioma, AH 6809 may reverse the molecular features caused by dysregulated TMED9 expression, thereby offsetting its mediated cancer-promoting effects. *p < 0.05, **p < 0.01, ***p < 0.001.

Recent studies have demonstrated that glioma stem cells play a critical role in glioma chemotherapy through a complex and intertwined signaling network (49). Notably, TMED9 exhibited a significant negative correlation with the tumor stemness score, RNAss, in gliomas (**Figure 10A**). Analysis utilizing the OCLR algorithm revealed that the TMED9 low-expression group had a higher stemness score (**Figure 10B**). These findings suggest that increased expression of TMED9 may inhibit the stemness characteristics of glioma cells, indicating that patients within the TMED9 low-expression group may exhibit heightened resistance to conventional anticancer treatments. Through an extensive literature review, we compiled multiple datasets related to glioma chemotherapy, which indicated significant differences in TMED9 expression when compared to a control group, including patients receiving first-line glioma treatment drugs such as temozolomide, disulfiram, and JQ1 (**Figures 10C–F**). Additionally, we estimated the IC50 values for 198 compounds from the GDSC database for each glioma patient and calculated the Pearson correlation coefficients with TMED9. Among these, 68 drugs exhibited a positive correlation with TMED9, whereas 99 drugs demonstrated a negative correlation. A bar chart presenting the top 20 drugs with the highest and lowest correlation coefficients is displayed in **Figure 10G**. Notably, among the top 20 negatively correlated drugs, ERK MAPK signaling inhibitors and PI3K/MTOR signaling inhibitors were predominantly utilized (**Figures 10H, I**). Furthermore, using the cMAP database, we identified potential small molecules and drugs (**Figure 10J**). Among these, the drug AH 6809 was recognized as a potential small molecule capable of rectifying the biological effects induced by dysregulated TMED9 expression in gliomas (**Figure 10K**).

## 4 Discussion

The accumulation of misfolded proteins within cells can lead to severe human protein diseases (50). TMED9, a member of the p24/emp24 domain-containing protein family, is involved in regulating intracellular protein trafficking and secretory pathways. It plays a crucial role in various cellular processes, including the vesicular trafficking of proteins, the quality control and degradation of misfolded proteins, and the biogenesis of autophagosomes (51). TMED9 regulates the transport of substances between the endoplasmic reticulum and the Golgi apparatus through interactions with coat protein I (COPI) and COPII coat proteins (52). During autophagy, TMED9 facilitates the formation of autophagosomes via membrane contacts mediated by SEC12, which is essential for cell survival under stress conditions (53). Furthermore, TMED9 is involved in regulating viral infections and immune responses, thus highlighting its multifunctionality and significance in cell biology and disease (54). In cancer, the overexpression of TMED9 correlates with the progression and poor prognosis of several cancers, including ovarian cancer, hepatocellular carcinoma, and breast cancer (8–10). Although the role of TMED9 in specific cancer types has been extensively studied, current research has predominantly focused on individual cancer types, lacking a comprehensive perspective needed to evaluate the expression patterns and functions of TMED9 across different cancer types. This limitation restricts our understanding of the mechanisms underlying TMED9’s role in pan-cancer and underscores the necessity for macroscopic pan-cancer studies to characterize the expression profile, genomic alterations, clinical prognostic value, and immunological features of the TMED9 gene.

Through multi-omics data analysis, we elucidated the expression landscape of TMED9 in pan-cancer. Our findings reveal significant upregulation of TMED9 mRNA and protein levels across multiple tumor tissues, consistent with prior research (55). The genetic alteration characteristics of TMED9 in pan-cancer offer crucial insights into its role in cancer pathology. Notably, TMED9 exhibited the highest mutation rate in KIRC, primarily due to amplification mutations. This observation aligns with previous genomic studies indicating that KIRC is often associated with increased gene copy number, which promotes tumor development (56). Different types of copy number variations have also been noted in other cancer types, such as ACC, BLCA, LUSC, and TGCT, further underscoring TMED9’s diverse role in various tumors. Additionally, we found a significant positive correlation between TMED9 copy number variations and its mRNA expression levels across almost all cancer types. This finding supports the established molecular mechanism in cancer, where increased expression of specific genes facilitates cancer cell survival and proliferation, which also explains the general upregulation of TMED9 during tumor progression. In our methylation analysis, TMED9 exhibited lower methylation levels in most tumors compared to normal tissues, with the exception of READ. This observation may relate to the demethylation phenomenon occurring in the tumor microenvironment, as the selective demethylation of DNA is closely associated with many cancers (57). Notably, in CHOL and UVM, TMED9 methylation levels significantly correlated with prognosis, suggesting that methylation status may serve as an important biomarker for predicting cancer outcomes. The positive correlation between TMED9 and genomic scores in various cancers further highlights its potential role in promoting chromosomal instability in tumor cells. Previous studies have underscored the significant role of genetic variation in tumorigenesis (58). Our eQTL-GWAS colocalization analysis identified specific single nucleotide polymorphisms (SNPs) of TMED9, such as rs6634 and rs34582406, which may share genetic variation across multiple tumor types, thereby providing novel genetic evidence for the tumor susceptibility associated with TMED9.

TMED9’s main roles in cells include regulating protein transport, secretion pathways, and the movement of substances between the endoplasmic reticulum and the Golgi apparatus. These functions are likely closely linked to the polarization and activation of immune cells. Therefore, we investigated the immunological characteristics of TMED9 across various cancers. Correlation analysis revealed a significant positive correlation between TMED9 and numerous immunomodulators, particularly MHC molecules. This suggests that the high expression of TMED9 confers advantages in antigen presentation and processing capabilities. This finding aligns with previous studies that indicate MHC molecules play a crucial role in the tumor immune response by influencing T cell activation and tumor-specific immune responses (59). Consequently, TMED9 may facilitate tumor recognition and clearance by the immune system through enhanced antigen presentation. The tumor microenvironment’s various immune subtypes are closely associated with patient prognosis. The TMED9 high expression group primarily correlates with the C1 subtype (wound healing type) and the C2 subtype (IFN-γ dominant type). In the tumor microenvironment, macrophages can promote tumorigenesis while also participating in anti-tumor immune responses. Immune cell infiltration analysis indicated that TMED9 exhibited a significant positive correlation with multiple immune cell types, particularly macrophages, underscoring the close relationship between TMED9 and immune regulation. TMED9 regulates secretory pathways (e.g., cytokine release, and antigen presentation) via ER-Golgi transport. By modulating MHC-I/II trafficking, it could influence macrophage polarization (e.g., M1/M2 balance), and T-cell activation. The interplay between pro-inflammatory and anti-inflammatory response mechanisms in tumors is crucial for regulating immune escape (60). TMED9 also demonstrates a significant positive correlation with several inflammation-related scores in various tumors, including GBM, LGG, SARC, and KIRC. These findings further underscore the essential role of TMED9 in the tumor immune microenvironment. Nevertheless, it is important to note that our pan-cancer analysis is primarily based on correlation analysis. Therefore, further mechanistic studies are needed to validate and support our conclusions.

As one of the most prevalent malignant tumors in the central nervous system, glioma has garnered significant research attention due to its complex molecular characteristics and immune environment (61). Given the notably high expression of TMED9 in glioma and its impact on patient prognosis, we focused on analyzing the relationship between TMED9 and glioma. In multiple glioma datasets, we observed that high TMED9 expression was significantly associated with poor prognosis. Multivariate Cox analysis further identified TMED9 as an independent prognostic factor for glioma, confirming its potential as a prognostic marker. Single-cell transcriptome and spatial transcriptomics analyses demonstrated that TMED9 was significantly upregulated in malignant cells and macrophages within glioma. This expression pattern suggests that TMED9 plays a crucial role in the glioma immune microenvironment, particularly in the interactions between tumor cells and immune cells. Additionally, we found that patients in the TMED9 high-expression group had shorter survival rates following immunotherapy and exhibited poorer responses to ICB. This result underscores that TMED9 may influence the efficacy of immunotherapy by modulating the tumor immune environment and its cellular infiltration characteristics. Functional enrichment analysis highlighted the correlation between TMED9 and glioma cell migration and invasion. Knocking down TMED9 in glioma cell lines significantly reduced the migration and invasion abilities of these cells, indicating that TMED9 may promote the biological characteristics of glioma. However, the current association between TMED9 and EMT is primarily based on correlational data and partial protein knockdown experiments. Rescue experiments, which would involve reintroducing TMED9 and assessing the expression of relevant EMT markers, have not yet been conducted. Notably, high TMED9 expression was significantly negatively correlated with the stemness score of glioma, suggesting that TMED9 may contribute to chemotherapy resistance in glioma by inhibiting tumor stem cell properties. This finding holds important clinical implications. Further drug sensitivity analysis revealed a strong association between TMED9 and chemotherapeutic drugs, including inhibitors of the ERK MAPK and PI3K/mTOR signaling pathways. However, additional laboratory or clinical validation is required to confirm the synergistic or antagonistic effects of these drugs.

In summary, our study provides new evidence supporting the potential of TMED9 as a cancer biomarker through a systematic analysis of its expression characteristics, genetic alterations, and biological functions in pan-cancer contexts. We also emphasize the significance of TMED9 findings in glioma research, showing that it not only promotes the invasiveness and migration of glioma cells but also impacts tumor responses to immunotherapy by modulating the immune microenvironment. Future studies should further investigate the specific mechanisms by which TMED9 operates in glioma. Concurrently, clinical trials to validate TMED9 as a therapeutic target may enhance glioma treatment efficacy. These efforts are crucial for improving patient prognosis and facilitating the development of new treatment models. We anticipate further discoveries regarding TMED9 in glioma research that will provide valuable insights for clinical applications.

## Data Availability

The original contributions presented in the study are included in the article/**Supplementary Material**. Further inquiries can be directed to the corresponding author/s.
