# Supplementary material for: TMED9: a potential therapeutic target and prognostic marker in glioma and its implications across pan-cancer contexts
Source: Front Immunol. 2025 Mar 7;16:1558881. doi: 10.3389/fimmu.2025.1558881 (PMC11925788; doi:10.3389/fimmu.2025.1558881)
Supplement: Supplementary file 1 [file DataSheet1.docx]

Supplementary Material

TMED9: A Potential Therapeutic Target and Prognostic Marker in Glioma and Its Implications Across Pan-Cancer Contexts

# Supplementary Table

**Supplementary Table 1.** **33 kinds of cancer detailed names**

| **Type** | **Description** |
| --- | --- |
| ACC | Adrenocortical carcinoma |
| BLCA | Bladder Urothelial Carcinoma |
| BRCA | Breast invasive carcinoma |
| CESC | Cervical squamous cell carcinoma and endocervical adenocarcinoma |
| CHOL | Cholangiocarcinoma |
| COAD | Colon adenocarcinoma |
| DLBC | Lymphoid Neoplasm Diffuse Large B-cell Lymphoma |
| ESCA | Esophageal carcinoma |
| GBM | Glioblastoma multiforme |
| HNSC | Head and Neck squamous cell carcinoma |
| KICH | Kidney Chromophobe |
| KIRC | Kidney renal clear cell carcinoma |
| KIRP | Kidney renal papillary cell carcinoma |
| LAML | Acute Myeloid Leukemia |
| LGG | Brain Lower Grade Glioma |
| LIHC | Liver hepatocellular carcinoma |
| LUAD | Lung adenocarcinoma |
| LUSC | Lung squamous cell carcinoma |
| MESO | Mesothelioma |
| OV | Ovarian serous cystadenocarcinoma |
| PAAD | Pancreatic adenocarcinoma |
| PCPG | Pheochromocytoma and Paraganglioma |
| PRAD | Prostate adenocarcinoma |
| READ | Rectum adenocarcinoma |
| SARC | Sarcoma |
| SKCM | Skin Cutaneous Melanoma |
| STAD | Stomach adenocarcinoma |
| TGCT | Testicular Germ Cell Tumors |
| THCA | Thyroid carcinoma |
| THYM | Thymoma |
| UCEC | Uterine Corpus Endometrial Carcinoma |
| UCS | Uterine Carcinosarcoma |
| UVM | Uveal Melanoma |

# Supplementary Figures


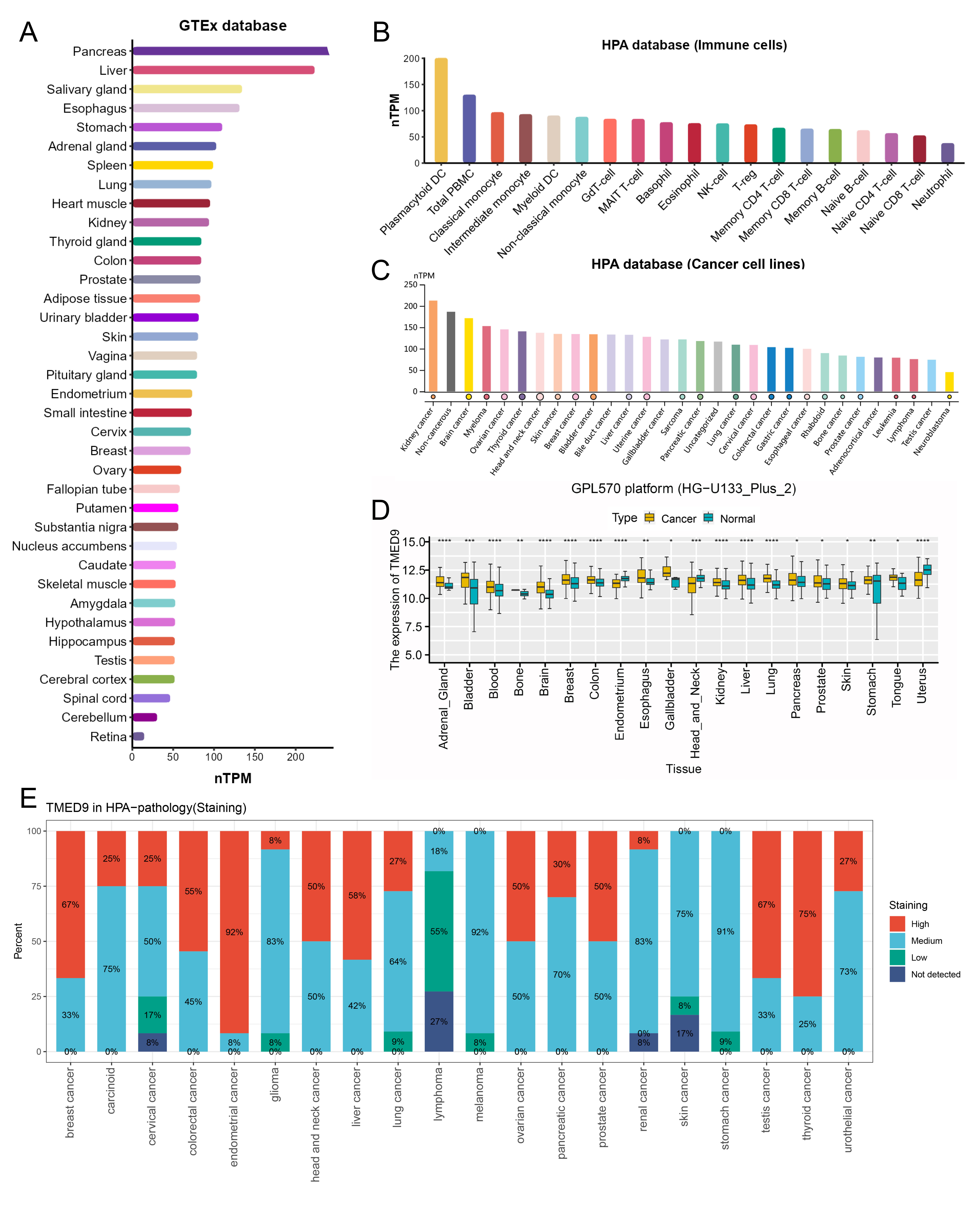


**Supplementary Figure 1.** (A) Evaluation of TED9 expression levels in normal human tissues using the GTEx database; (B) Evaluation of TED9 expression levels in immune cells based on the HPA database; (C) Evaluation of TED9 expression levels in tumor cell lines based on the HPA database;(D) GENT2 validates the differential expression of TMED9 between tumor samples and normal samples; (E) Statistics of TMED9 immunohistochemistry staining results in the HPA database.

**
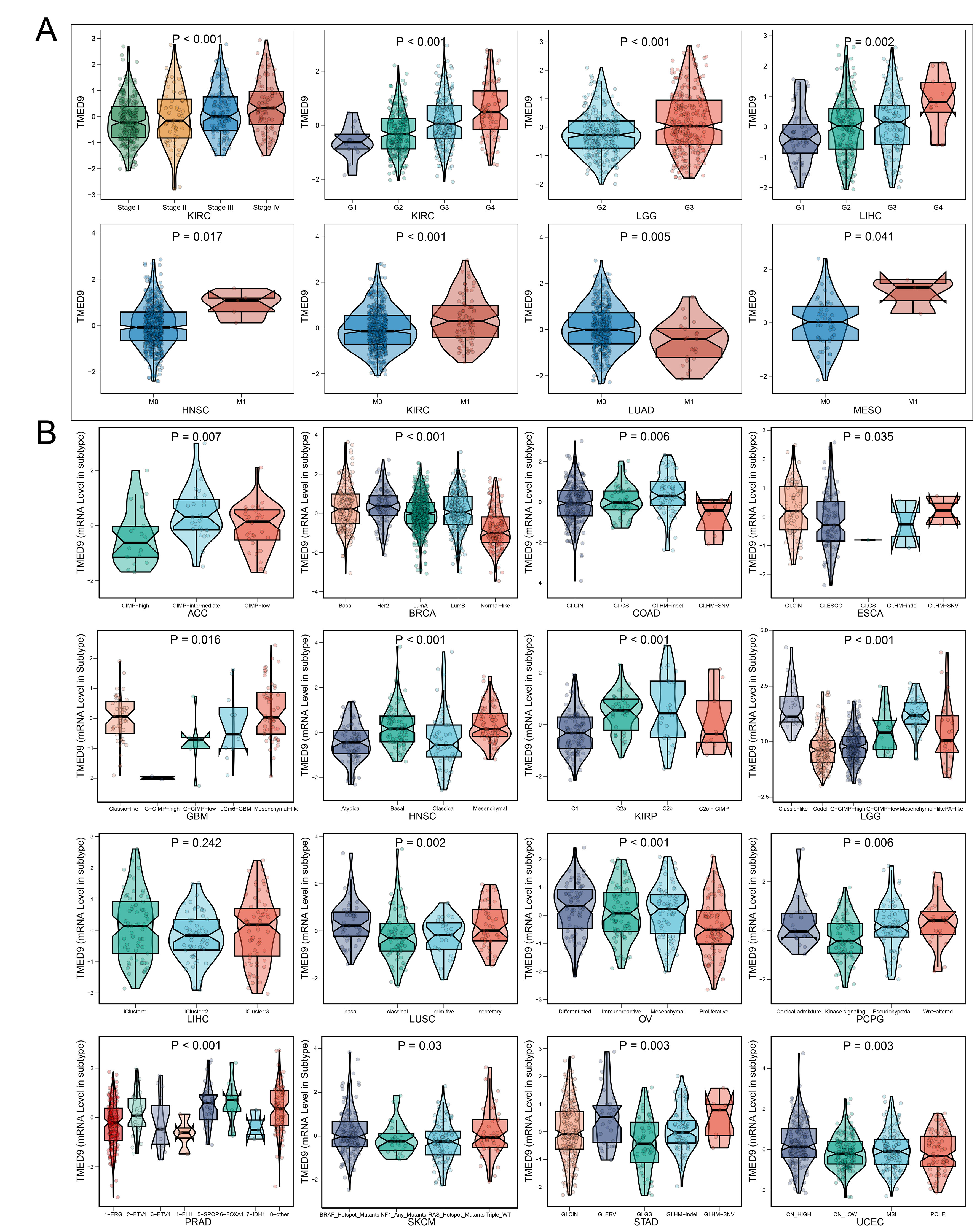
**

**Supplementary Figure 2.** (A) The relationship between TMED9 and clinical stage in pan-cancer; (B) Relationship between TMED9 and molecular subtypes in pan-cancer.

**

**

**Supplementary Figure 3.** (A-I) Visualization of TMED9 pan-cancer eQTL-GWAS co-localization analysis results using gassocplot2 package.

**
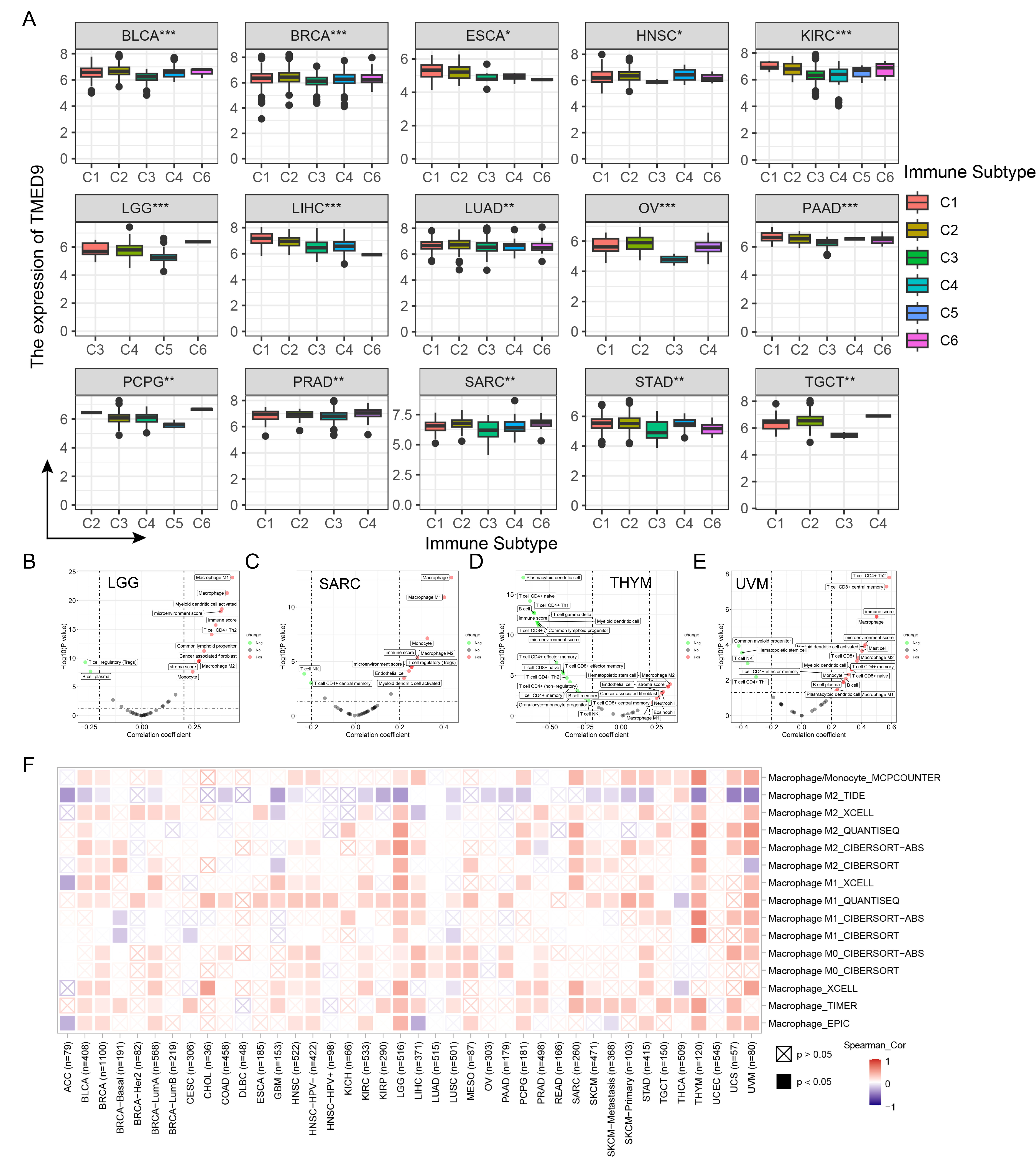
**

**Supplementary Figure 4.** (A) TMED9 expression levels among different immune subtypes in pan-cancer; (B-E) Correlation between TMED9 and immune cell infiltration in LGG, SARC, THYM, and UVM; (F) Evaluation of the correlation between TMED9 and macrophage infiltration in pan-cancer based on the TIMER2.0 online database.

**
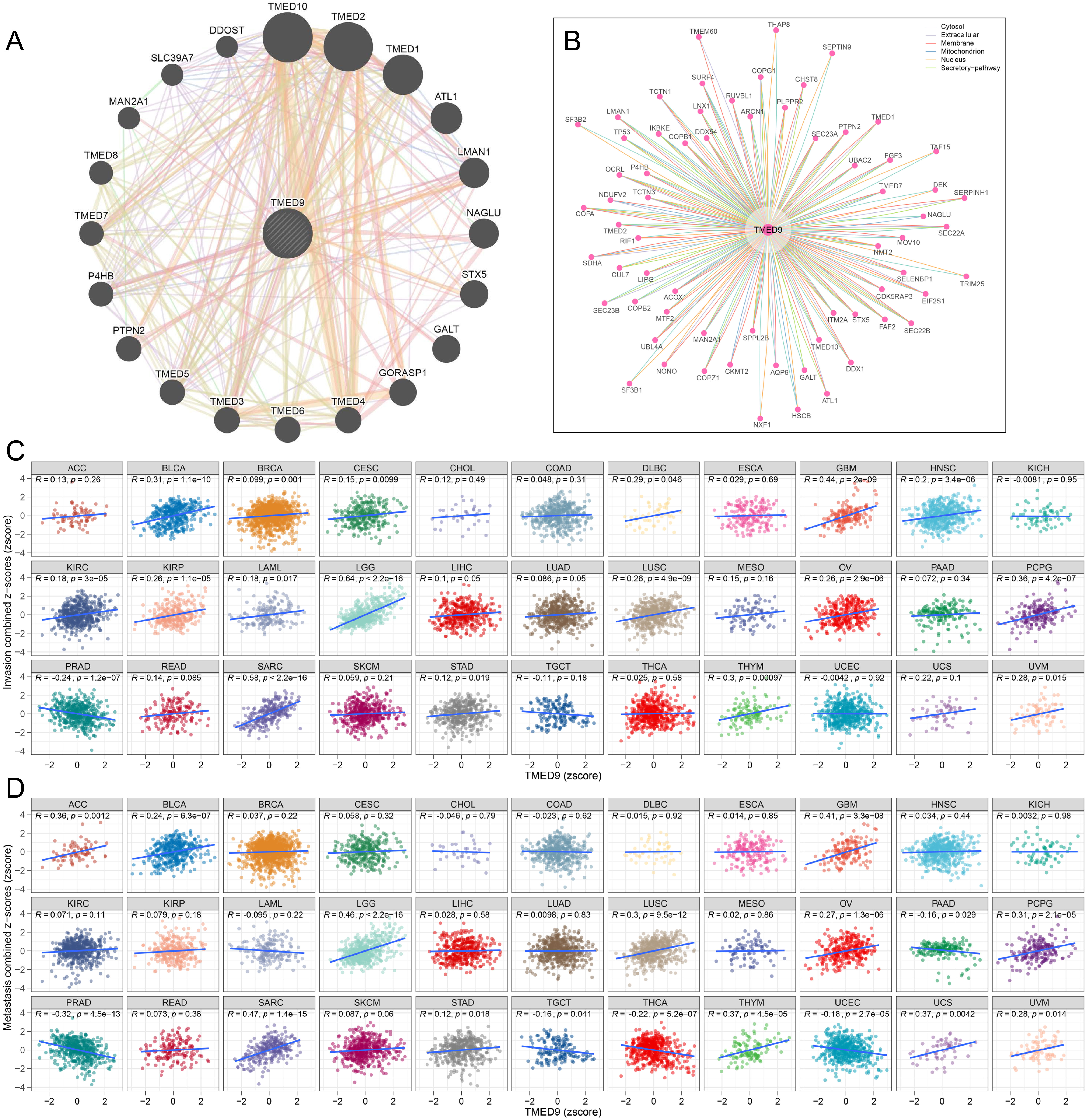
**

**Supplementary Figure 5.** (A) Evaluation of TMED9 gene interaction network using GeneMANIA database; (B) Evaluation of TMED9 protein interaction network using the comPPI database; (C) Pearson correlation analysis between pan-cancer invasion signature score and TMED9; (D) Pearson correlation analysis between pan-cancer metastasis signature score and TMED9.

**

**

**Supplementary Figure 6.** (A-L) ROC analysis of TMED9 expression in the diagnosis of tumor and normal groups.

**

**

**Supplementary Figure 7.** (A-T) Kaplan-Meier survival analysis to evaluate the correlation between TMED9 expression and pan-cancer overall survival and progression free interval

**

**

**Supplementary Figure 8.** (A-Q) Kaplan-Meier survival analysis to evaluate the correlation between TMED9 expression and pan-cancer disease-specific survival and disease-free interval.

**
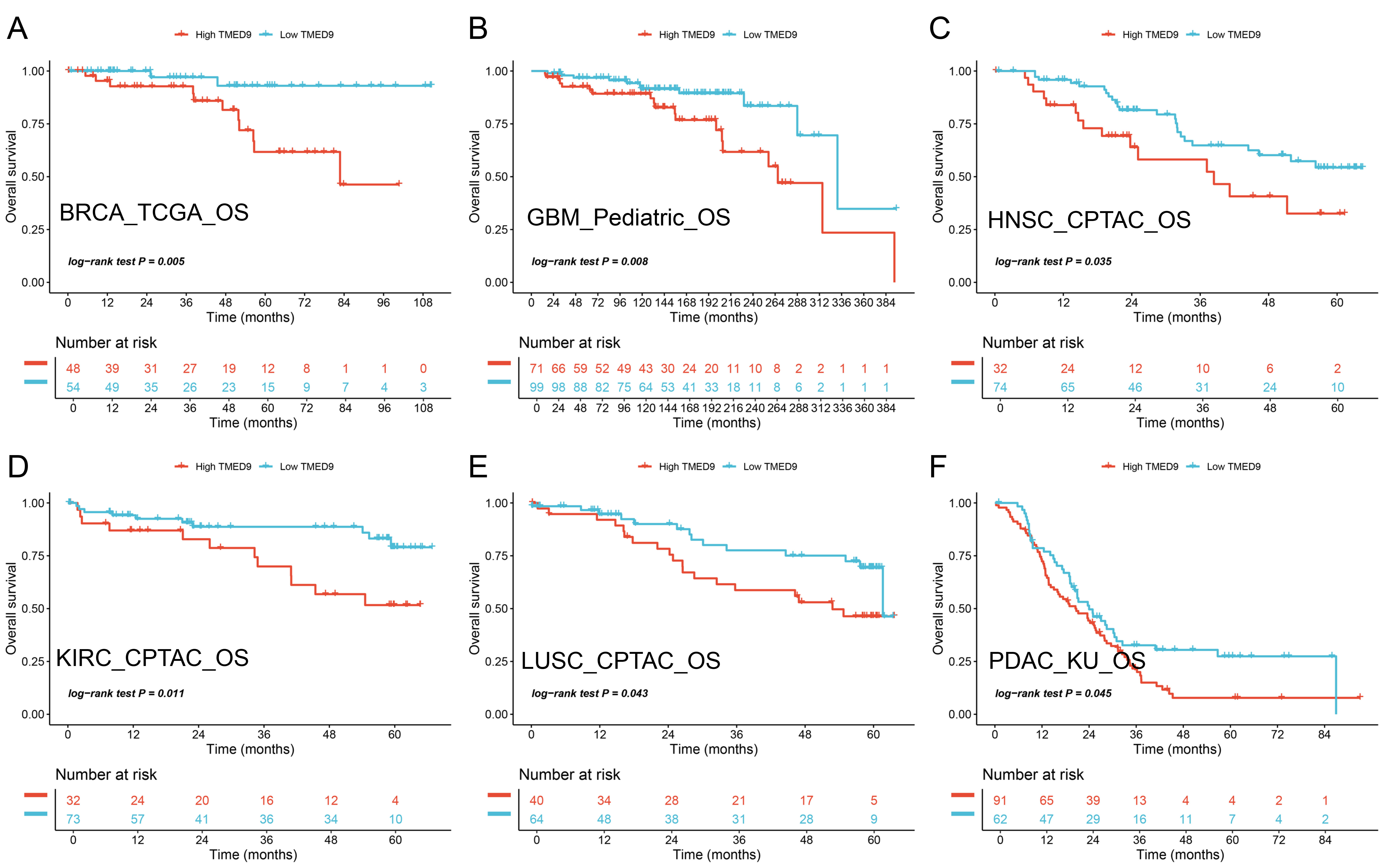
**

**Supplementary Figure 9.** (A-F) Kaplan-Meier survival analysis based on proteomic data evaluated the correlation between TMED9 expression and overall survival in multiple cancer types.

**
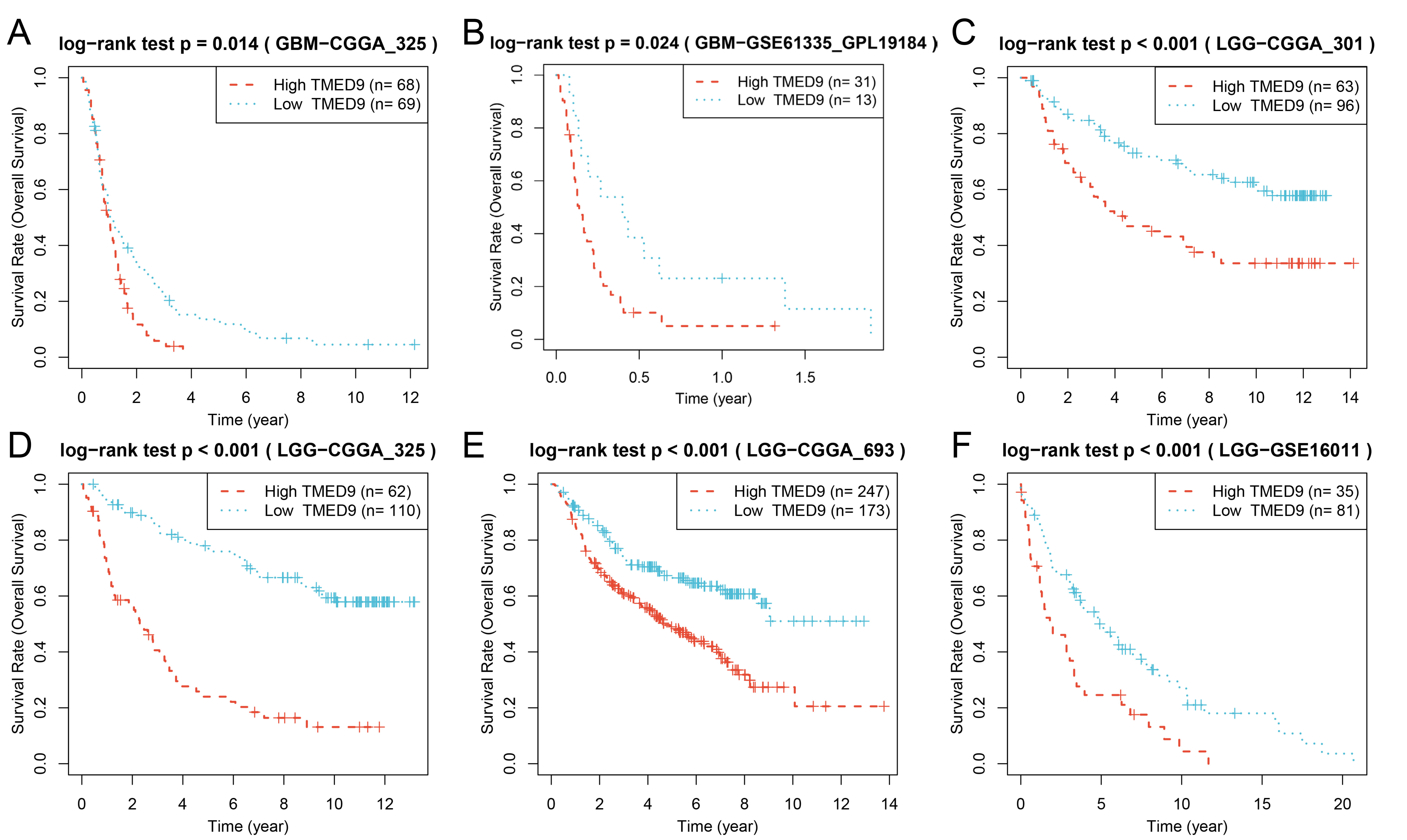
**

**Supplementary Figure 10.** (A-F) Multiple external glioma datasets validate the association between TMED9 and glioma prognosis.

**
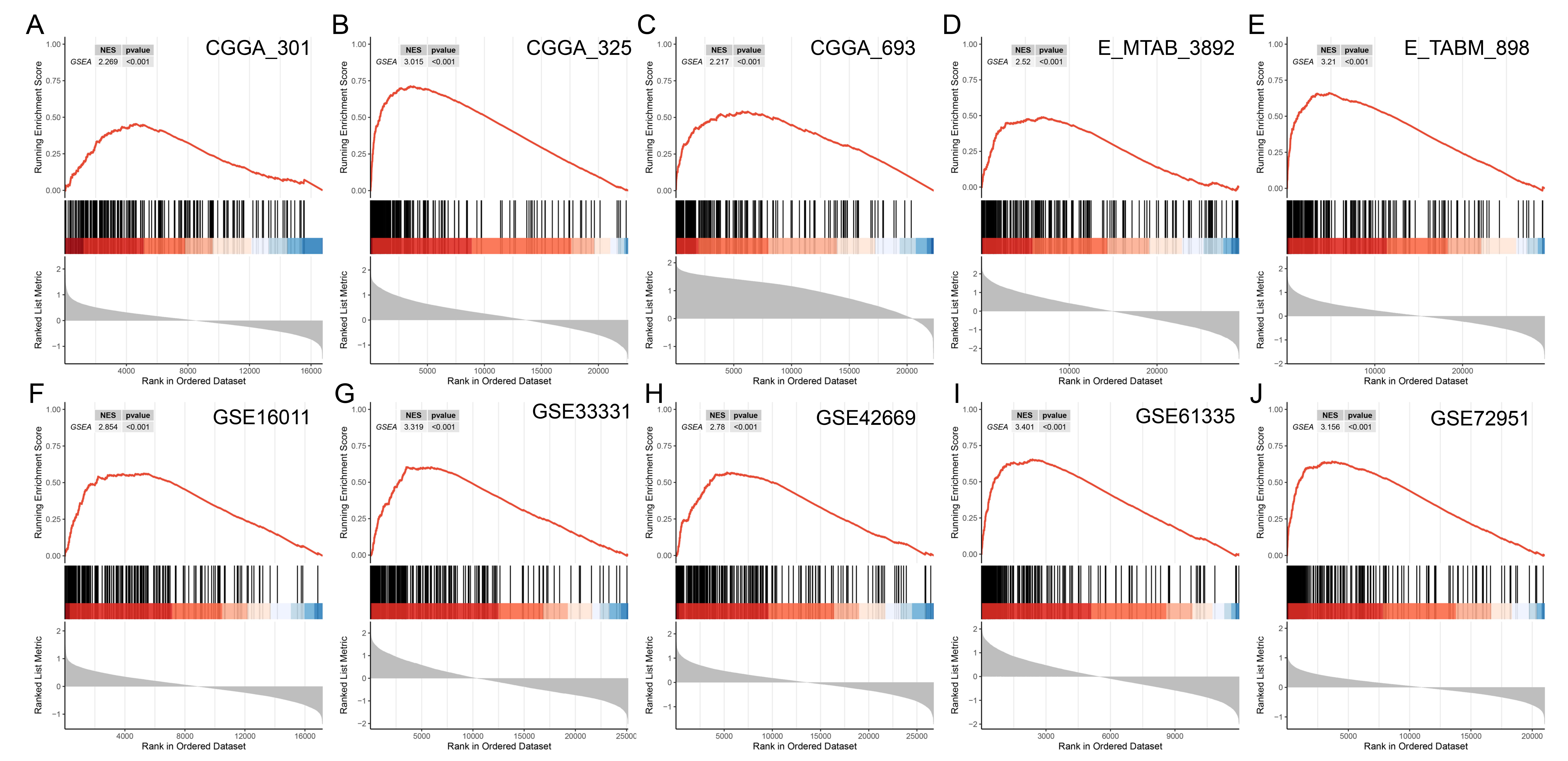
**

**Supplementary Figure 11.** (A-J) GSEA results of multiple external glioma datasets validated the association between TMED9 and the glioma EMT pathway.
